# CL-316243 facilitates stable atherosclerotic plaque phenotypes in association with suppression of perivascular adipose tissue ferroptosis via upregulating C/EBPβ

**DOI:** 10.1016/j.redox.2026.104259

**Published:** 2026-06-13

**Authors:** Yuanqing Jiang, Yi Li, Kefan Ma, Suxiang Guo, Nachuan Liao, Jiayi Zhou, Junbo Chen, Ruizhe Ren, Yaohui Kou, Jinying Li, He Liu, Yang Wei, Xiaofei Zhou, Linge Fan, Lingfeng Qin, Haige Zhao, Ying Xiao, Luyang Yu, Zhen Ge, Cong Qiu

**Affiliations:** a MOE Laboratory of Biosystems Homeostasis & Protection, Zhejiang University-Lishui Joint Innovation Center for Life and Health of College of Life Sciences, Zhejiang University, Hangzhou, Zhejiang, China; b Key Laboratory of Neuropsychiatric Drug Research of Zhejiang Province, School of Pharmaceutical Sciences, Hangzhou Medical College, Hangzhou, Zhejiang, 310013, China; c Department of Cardiovascular Surgery, First Affiliated Hospital, Zhejiang University School of Medicine, Hangzhou, 310003, China; d Sir Run Run Shaw Hospital, School of Medicine, Zhejiang University, Hangzhou, China; e Cardiovascular Research Center, Interdepartmental Program in Vascular Biology and Therapeutics, Department of Internal Medicine, Yale University School of Medicine, New Haven, CT, 06520, USA

**Keywords:** Atherosclerosis, Stable plaque phenotype, CL-316243, Arterial perivascular adipose tissue, Ferroptosis, C/EBPβ

## Abstract

Atherosclerotic plaque rupture is the leading cause of most acute cardiovascular events and death. However, effective treatment strategies targeting plaque stability remain elusive. Perivascular adipose tissue (PVAT) dysfunction is increasingly recognized as a contributor to vulnerable plaque, implying that elucidating and targeting its underlying mechanism could represent a novel therapeutic strategy for atherosclerosis. This study identifies ferroptosis as a critical pathological feature in PVAT of atherosclerotic artery, establishing a previously unrecognized link between PVAT dysfunction and ferroptosis. The β3-adrenoceptor agonist CL-316243 (CL) significantly suppressed ferroptotic markers in both cultured adipocytes and atherosclerotic PVAT. Furthermore, CL treatment reduced PVAT inflammation and dysfunction, as shown by histological, gene expression, and functional analyses, thereby facilitating stable plaque phenotypes, reflected in reduced necrotic core size, lower CD68 and MMP expression, and increased collagen content. Notably, the decline in ferroptosis markers correlated significantly with these phenotypic improvements. Mechanistically, C/EBPβ was identified as a direct transcriptional regulator of *GPX4* through CUT&Tag and dual-luciferase reporter assays, by which the critical promoter binding motif was also identified. Subsequently, CL was shown to upregulate GPX4 via C/EBPβ, and this effect was abolished by *Cebpb* knockdown. Further analysis indicated that CL-induced upregulation of C/EBPβ depends on the cAMP-PKA-CREB pathway. Finally, *Cebpb* knockdown abolished the anti-ferroptosis effect of CL, confirming C/EBPβ as a critical component of this mechanism. Our study reveals that CL stabilizes atherosclerotic plaques by suppressing PVAT ferroptosis through the C/EBPβ-GPX4 axis, uncovering novel therapeutic targets and providing a potential treatment strategy for atherosclerotic diseases.

## Highlights

• CL-316243 promotes stable atherosclerotic plaque phenotypes by inhibiting ferroptosis-induced dysfunction of perivascular adipose tissue.
• C/EBPβ is identified as a novel transcriptional regulator of GPX4 in adipocytes.
• CL-316243 upregulates GPX4 through the cAMP-PKA-CREB-C/EBPβ pathway.
• Inhibition of PVAT ferroptosis may represent a potential strategy to facilitate stable plaque phenotypes in established atherosclerosis.

## Graphical abstract

**Figure:**
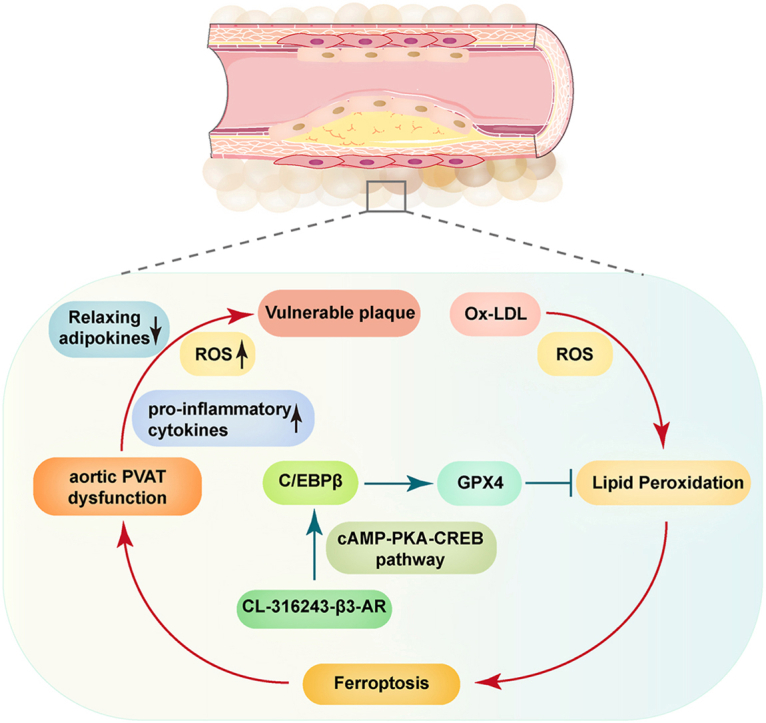

## 1 Introduction

Cardiovascular disease (CVD) remains the leading cause of mortality worldwide. A key risk factor for CVD is atherosclerosis, which can progress to life-threatening diseases such as myocardial infarction and stroke [1]. Vulnerable plaques, characterized by lipid-rich, large necrotic core, and thinner fibrous caps, are present in late-stage atherosclerosis. These plaques have been confirmed as an independent predictor of acute cardiovascular events due to their high risk of sudden rupture and thrombosis [2,3]. Although antiplatelet and plasma lipid-lowering treatments have been made progress in the prevention and treatment of CVDs in recent years, atherosclerotic plaque rupture remains the primary pathogenic mechanism of CVDs. Therefore, elucidating novel mechanisms underlying atherosclerotic plaque rupture and developing innovative therapeutic strategies are pivotal for preventing acute CVD events and improving clinical outcomes.

Most blood vessels are surrounded by a functionally specialized aggregate of adipose tissue known as perivascular adipose tissue (PVAT), which plays an important role in CVD [[4], [5], [6], [7]]. For a long time, PVAT was considered to serve only structural and vessel-supporting purposes; however, its endocrine, paracrine and metabolic functions have been ignored. The absence of PVAT can increase the risk of atherosclerosis in mice [8,9]. During atherosclerosis progression, high-energy intake promotes chronic local proinflammatory and atherosclerotic pathological conditions in vessels, followed by PVAT dysfunction, such as an increase in mass, whitening, metabolic dysfunction, and the release of adipose cytokines to promote macrophage infiltration and proinflammatory cytokine release in the aortic perivascular area, thus exacerbating vascular inflammation and aortic lesions in aortic wall tissue [[9], [10], [11]]. Notably, in the advanced stage of atherosclerosis, increased levels of pro-inflammatory adipose-derived cytokines were detected in aortic PVAT [12]. Therefore, PVAT dysfunction may play a direct role in triggering vulnerable plaque through the “outside-to-inside” pathway [13]. Revealing the novel regulatory mechanism of PVAT and targeting it may be a potential new therapeutic strategy for atherosclerosis compared to traditional drug therapies.

Ferroptosis is induced by iron (Fe)-dependent lipid peroxidation and is accompanied by inflammatory cytokine synthesis and secretion [14,15], which promotes local tissue inflammation and dysfunction. Recently, studies have demonstrated that ferroptosis in plaque cells, such as endothelial cells, macrophages, and vascular smooth muscle cells, play a critical role in promoting atherosclerosis [[16], [17], [18], [19], [20], [21]]. In particular, macrophage ferroptosis was indicated in involving plaque vulnerability [22]. In addition, ferroptosis inhibition in endothelial cells and macrophages suppressed atherosclerosis [[23], [24], [25]]. However, the role of ferroptosis in artery PVAT during atherogenesis remains unknown, despite evidence that oxidative stress in PVAT elevates local reactive oxygen species (ROS) production [26,27], which is a critical driver of lipid peroxidation and ferroptosis [28,29].

CL-316243 (CL) is a selective β3-adrenoceptor (β3-AR) agonist, which has been found to improve insulin action and fat oxidation in clinical trials [30]. Moreover, it plays a significant role in promoting mitochondrial synthesis, thermogenesis, energy expenditure and ROS elimination [31,32], indicating it may be a novel ferroptosis inhibitor. In addition, the β3-AR is located mainly in the adipose tissue and is involved in the regulation of lipolysis and thermogenesis. Therefore, CL may act as a novel therapeutic strategy to suppress arterial PVAT ferroptosis through β3-AR-mediated signaling, thereby stabilizing atherosclerotic plaques. However, the precise role and underlying mechanisms of CL in PVAT ferroptosis and stable plaque phenotypes remain incompletely understood.

Our study reveals PVAT ferroptosis as a previously unrecognized mechanism linking adipose dysfunction to atherosclerosis. Moreover, we demonstrate that CL suppresses ferroptosis *in vitro* and *in vivo*. Further study showed that CL protected arterial PVAT from dysfunction during atherogenesis and stabilized atherosclerotic plaques. Mechanistically, CL inhibited mitochondrial dysfunction and excessive lipid peroxide levels by transcriptionally promoting glutathione peroxidase 4 (GPX4) expression through activation of the β3-AR-cAMP-PKA-CCAAT/enhancer binding protein beta (C/EBPβ) axis. Importantly, C/EBPβ is identified here as a novel transcriptional regulator of GPX4, offering a new molecular target for therapeutic intervention. Our study highlights the potential of CL-induced elimination of arterial PVAT ferroptosis as a strong candidate for the treatment of atherosclerotic diseases.

## 2 Methods

### 2.1 Mice and treatments

Four-week-old male C57BL/6 mice (weighing 15-18 g for adipocyte isolation), 8-week-old male *Apoe*^*−/−*^ mice on a C57BL/6 background (weighing 18-23 g, Shanghai SLAC Laboratory Animal Co., Shanghai, China) were housed in the animal facilities at Zhejiang University in a humidity- and temperature-controlled environment (55 ± 5%, 22 ± 2°C) with a 12 h light-dark cycle and no pathogenic microorganisms. All researches were following the Guide for the Care and Use of Laboratory Animals published by the US National Institutes of Health (NIH Publication, 8th Edition, 16 2011), and were approved by the Institutional Animal Care and Use Committee of Zhejiang University (Approved code: ZJU20230181).

For the established atherosclerosis model, *Apoe*^*−/−*^ mice were assigned to 16 weeks of a high-fat diet (HFD, Research Diets Inc., USA, #D12108) containing 40 kcal by percent saturated fat and 1.25% cholesterol. To examine the effect of the β3-adrenergic receptor agonist CL-316243 (CL, Med Chem Express (MCE), China, #HY-116771A) on ferroptosis and atherosclerotic plaque phenotypes, the mice were randomly divided into two groups: vehicle control group (saline) and 1 mg/kg CL treatment group (n = 6 per group for each assay). After 12 weeks of HFD feeding, the control and treatment groups were intraperitoneally administered the indicated treatments (i.p.) daily between 9:30 to 10:30 a.m. for 4 consecutive weeks.

All groups of mice were anaesthetized by isoflurane inhalation (3%) and euthanized by exsanguination (RWD, R510-22-10) before sample harvest. Mice were randomly assigned to groups, and tissue collection and image quantification were performed by blinded, independent personnel. Sick animals or animals that died prior to a time point were not included in the final analysis.

### 2.2 PVAT bulk RNA sequencing

RNA sequencing was performed on freshly isolated perivascular adipose tissue (PVAT) from control group (*Apoe*^*−/−*^ mice with 16 weeks of normal diet feeding) and atherosclerotic group (*Apoe*^*−/−*^ mice with 16 weeks of HFD feeding), each of which contained five biological replicates. After isolation, PVAT was immediately placed in ice-cold, sterile phosphate-buffered saline (PBS) supplemented with penicillin–streptomycin (P/S) and washed 2 times to remove residual blood and contaminants. Each sample was then extracted using 300 μL RNAlater reagent (Ambion, #AM7021) followed by RNA sequencing (LC-Bio Technologies (Hangzhou) Co., Ltd). StringTie and ballgown were used to estimate the expression levels of all transcripts and perform expression abundance for mRNAs by calculating FPKM (fragment per kilobase of transcript per million mapped reads) value. Genes differential expression analysis was performed by DESeq2 software between two different groups. The genes with the parameter of false discovery rate (FDR) below 0.05 and absolute fold change ≥1.5 were considered differentially expressed genes. The heatmap were generated using OmicStudio (https://www.omicstudio.cn/cell). Gene Ontology (GO) and Kyoto Encyclopedia of Genes and Genomes (KEGG) analysis of differentially expressed genes, as well as Gene Set Enrichment Analysis (GSEA) analysis, were performed using the OmicStucio tools at https://www.omicstudio.cn/tool.

### 2.3 Ex vivo culture and pharmacological treatment of PVAT

Freshly isolated PVAT was immediately placed in ice-cold, sterile PBS supplemented with P/S and washed 2 times to remove residual blood and contaminants. The tissue was then transferred to a new tube, accurately weighed, and distributed into 24-well plates at a standardized mass of 20 mg per well, followed by an additional 2 washes with sterile PBS (+P/S) [33,34]. PVAT explants were cultured in DMEM containing 5% fetal bovine serum (FBS) for 24 h to allow recovery and stabilization. Thereafter, they were treated in 5% FBS–DMEM with the following agents as specified in the figure legends: oxidized LDL (Ox-LDL) (Yeasen, China, #20605ES05, 75 μg/mL), CL-316243 (1 μM), Ferrostatin-1 (Fer-1) (MCE, China, #HY-100579, 5 μM), RSL3 (MCE, China, #HY-100218A, 5 μM), or N-acetylcysteine (NAC) (MCE, China, #HY-B0215, 5 μM) for 24 or 48 h. Following treatment, conditioned media were collected and centrifuged at 5000 rpm for 5 min to remove cellular debris; the resulting supernatants were stored at −80°C for subsequent quantification of inflammatory cytokines [35,36]. Tissues were used for further investigations. All experimental conditions and group assignments are detailed in the corresponding figure legends.

### 2.4 RNA isolation and qPCR assays

Adipose tissues or adipocytes were homogenized and lysed by using Universal RNA extraction Kit (Accurate Biology, China, #AG21022). mRNA was isolated and reverse transcribed into cDNA using the *Evo M-MLV* RT Master Mix (Accurate Biology, China, #AG11706). The qPCR was performed using SYBR Green *Pro Taq* HS Premix (Accurate Biology, China, #AG11701) according to the instruction protocols. Target gene mRNA expression was normalized to internal control GAPDH. The relative mRNA level was quantified by 2–ΔΔCt method from at least 3 or more independent experiments. Primer sequences of tested genes were listed in Table S1.

### 2.5 TUNEL assay

TUNEL was assessed by the TUNEL Apoptosis Detection kit (Yeasen, China, #40307ES50), according to the instruction protocols. Briefly, PVAT paraffin sections were deparaffinized and rehydrated at room temperature, followed by permeabilization with Proteinase K solution for 20 min. The samples were then incubated with YSFluor™ 488-12-dUTP Labeling Mix at 37°C for 1 h. Afterward, nuclei were counterstained with DAPI for 10 min. Slides were then mounted and imaged under a fluorescence microscope. For cultured cells, samples were mixed with YSFluor™ 488-12-dUTP Labeling Mix reagent and incubated at 37°C for 40 min in the dark. After washing twice with PBS, the positive fluorescence intensity was immediately imaged using a Nikon fluorescence microscope and analyzed with Image J.

### 2.6 Immunohistochemistry (IHC) analysis

IHC staining was performed using a rabbit ABC Staining kit (Neobioscience, China, #VAR100) according to the protocol provided by the manufacturer. Briefly, paraffin sections of PVAT were deparaffinized and hydrated followed by antigen retrieval with microwaved 10 mM Sodium Citrate buffer pH 6.0 or Tris/EDTA buffer pH 9.0 for 24 min prior to blocking and staining. Then sections were incubated in 3% hydrogen peroxide for 15 min to quench endogenous peroxidase. After that, sections were further permeabilized using 0.3% Triton X-100 in PBS for 10 min and incubated in 2% BSA and 5% blocking horse serum in PBS buffer for 1 h at room temperature and then incubated with anti-4-hydroxynonenal (4-HNE) (Abcam, ab48506, 1:100), anti-UCP1 (Abcam, #ab234430, IHC: 1:400), or anti-GPX4 (Abclonal, #A13309, IHC: 1:100) primary antibodies overnight at 4°C. In the next day, after washing, the sections were incubated with HRP-conjugated goat anti-rabbit IgG (H + L) for 1 h at room temperature and color rendered with DAB (Boster, #AR1027). The nuclei were stained with hematoxylin, dehydrated and mounted. The histopathological changes were observed under a light microscope and the IHC results were quantified by using ImageJ software with plugin IHC toolbox, and the mean IHC-positive intensity of corresponding antibodies staining was determined by average optical density (AOD).

### 2.7 Determination of malondialdehyde (MDA) and glutathione (GSH)/oxidized glutathione (GSSG) ratio

The MDA and GSH/GSSG ratio *in vivo* and *in vitro* were determined by the Lipid Peroxidation MDA Assay kit (Beyotime, #S0131S) and GSH and GSSG Assay kit (Beyotime, #S0053) according to the instruction protocols.

### 2.8 Fe^2+^ determination

The Fe^2+^ content was measured using a Ferrous Ion Content Assay Kit (Solarbio, #BC5414) according to the manufacturer's instructions. Briefly, samples were homogenized by extraction solution on ice, followed by centrifugation at 10,000 × g for 10 min at 4°C. The supernatant was collected, and Reagent I was added, mixed thoroughly, and incubated at 37°C for 10 min. Chloroform was then added, and the mixture was vortexed vigorously for 5 min. After centrifugation at 12,000 × g for 10 min at room temperature, the upper aqueous phase was transferred to a 96-well plate. The absorbance at 593 nm was measured using a microplate reader.

### 2.9 Isolation, culture, adipogenic induction of preadipocytes from PVAT-derived stromal vascular fraction (SVF), and cell treatment

Arterial PVAT-derived SV fractions (SVFs) were isolated from C57BL/6 mice as described previously [37]. Briefly, the tissues were washed, minced, and then digested for 45 min at 37°C in KRBS buffer (Shenzhen Ziker Biological Technology, China, #ZK-L0513) containing 2% fatty acid-free bovine serum albumin (BSA), 8 units/mL collagenase I (Sigma‒Aldrich Fine Chemical, USA, #SCR103) and 10 mM CaCl_2_ with shaking at 100 cycles/min. The suspension was filtered through 100-μm and 40-μm nylon filters and centrifuged at 1000 ×g for 5 min at room temperature. The pellets were suspended in erythrocyte lysis buffer (Beyotime Biotechnology, China, #C3702) for 1 min and centrifuged at 1000 ×g for 5 min at room temperature. The pellets were resuspended in culture medium (DMEM supplemented with 10% FBS and P/S) and plated on collagen-coated dishes.

The cells were plated onto collagen-coated dishes and grown to confluence in culture medium and differentiated into adipocytes [38]. Briefly, the cells were cultured for 48 h when achieving confluent and exposed to the adipogenic cocktail containing 20 μM insulin (Sigma-Aldrich, USA, #I5523), 0.5 mM IBMX (Sigma-Aldrich, USA, #I7018), 5 μM dexamethasone (Sigma-Aldrich, USA, #D4902), 1 nM *3,3′,5-Triiodo-**l**-thyronine* (T3, Sigma-Aldrich, USA, #T2877), and 200 nM *indomethacin* (Sigma-Aldrich; #I8280) in SVF culture medium for another 4 days. For every 48 h, the medium was refreshed. After 4 days induction, cells were maintained in culture medium containing 20 μM insulin and 1 nM T3 for another 4 days until the cells were used for experiments.

For pharmacological treatments, cells were exposed to Ox-LDL (150 μg/mL), Fer-1 (5 μM), CL (1 μM), or PKA inhibitor Rp-cAMPS (MCE, China, #HY-100530A, 20 μM) for the indicated conditions. For phosphorylation studies, following an initial 24-h culture in DMEM supplemented with 10% FBS, the medium was replaced with DMEM containing 0.5% FBS for an overnight serum starvation period followed by indicated treatments.

For small interfering RNA (siRNA) transfection, the siRNA against *Cebpb* and *Ppara* (*Cebpb*-siRNA: 5′-CCCUGCGGAACUUGUUCAAGCAGCUdAdT-3′, *Ppara*-siRNA: 5′-GAUCGGAGCUGCAAGAUUCdAdT-3′) as well as their negative controls (NC-siRNA: 5′-AATTCTCCGAACGTGTCACGTdAdT-3′) were acquired from Sangon Biotech (Shanghai, China). Following by the instruction's protocol, 50 nM of the above siRNAs were transfected into cells by using Lipofectamine RNA iMAX (Invitrogen, USA, #13778075) at day 5 of differentiation according to the instruction protocols. After 72 h transfection, cells are used for subsequent experiments.

All experimental conditions and group assignments are detailed in the corresponding figure legends.

### 2.10 Cell viability assay

Cell viability was measured by cell-counting kit-8 (CCK8) assay (Beyotime Biotechnology, China, #C0038) after treatment according to the instruction protocols.

### 2.11 Mitochondrial membrane potential assay

Mitochondrial membrane potential was detected by a mitochondrial-specific dual fluorescence probe, JC-1 (YEASEN Biotechnology, China, #40705ES03). Briefly, treated cells were washed with PBS and labeled with JC-1 (5 μg/mL), followed by incubation for 40 min at 37°C avoiding light. After that, the cells were washed with PBS for three times and resuspended with 1 mL PBS. The fluorescence activity was analyzed by flow cytometry.

### 2.12 Reactive oxygen species assays

The mitochondrial and lipid ROS levels in arterial PVAT tissue and differentiated adipocytes were measured by the mitochondrial ROS indicator MitoSOX red (YEASEN, China, #40778ES50) and C11-BODIPY^581/591^ (Abclonal, China, #RM02821), respectively, according to the instruction protocols. The positive fluorescence signal was immediately analyzed by flow cytometry and analyzed by FlowJo.

### 2.13 Transmission electron microscopy (TEM)

After treatments, the cells were rinsed in PBS and then fixed in 2.5% glutaraldehyde PBS buffer for 2 days. After washing with PBS three times, samples were fixed in 1% OsO_4_ (dilution in PBS) for 1 h. Samples were washed in ddH_2_O, stained in uranyl acetate for 30 min, dehydrated in a gradient of ethanol and acetone, permeated by embedding medium containing acetone (1:1), and finally embedded in epoxy resin. The ultrathin electron-stained sections were observed under an electron microscope (Philips-FEI, Tecnai T10, NL) with voltage setting at 100 kV. Micrographs of randomly selected mitochondria and cell were obtained at a final magnification of ×30000.

### 2.14 ELISA

The levels of TNF-α, IL-1α and IL-6 in collected conditional medium were detected by corresponding kits (mouse TNF-α Kit, Abcam, UK, #ab208348; mouse IL-1α Kit, Abcam, UK, #ab199076; mouse IL-6 Kit, Abcam, UK, #ab222503) according to the manufacturers’ instructions.

For cyclic adenosine monophosphate (cAMP) detection, the PVAT lysate was prepared in 0.1 M HCL, and diluted with assay buffer. And then cAMP levels of PVAT were detect by cAMP ELISA Kit (Abcam, UK, #ab290713) according to the manufacturers’ instructions.

### 2.15 Western blotting (WB) analysis

For tissue lysates, isolated adipose tissue was homogenized in radioimmunoprecipitation assay (RIPA) lysis buffer (Beyotime Biotechnology, #P0013C) supplemented with protease inhibitor cocktail (Roche, Germany, #4693132001). For cell lysates, cells were harvested with cold lysis buffer containing 50 mM Tris-HCl, 150 mM NaCl, 0.6% Triton X-100, 1 mM EDTA, 1 mM EGTA and 1X cocktail protease inhibitor at pH 7.5. The tissue homogenates and cell lysates were then centrifuged at 12,000 rpm for 20 min at 4°C, and the supernatant was used for determination. Total protein concentrations were determined by the BCA protein assay Kit (FDbio, China, #FD2001). Equal amounts of protein were loaded and separated by 8% or 10% or 12% sodium dodecyl sulfate-polyacrylamide gel electrophoresis (SDS-PAGE) and transferred to polyvinylidene difluoride membranes followed by blocking with 5% milk in TBST for 1 h at room temperature. Membranes were then incubated with corresponding primary antibodies overnight at 4°C. The primary antibodies including: anti-UCP1 (Abcam, UK, #ab234430, 1:1000), anti-PGC-1α (Abclonal, China, #A11971, 1:1000), anti-β-Actin (Abclonal, China, #AC006, 1:10000), anti-GPX4 (Abclonal, China, #A13309, 1:1000), anti-C/EBPβ (Abclonal, China, #A0711, 1:1000), anti-PPARα (Abclonal, China, #A18252, 1:1000), anti-PKA (Santa Cruz, USA, #sc-365615, 1:500), anti-*p*-PKA (Thr197) (Abcam, UK, #75991, 1:500), anti-CREB (Abclonal, China, #A27461, 1:1000), and anti-*p*-CREB (Ser133) (Cell Signaling Technology, USA, #9198, 1:500), anti-Vinculin (Cell Signaling Technology, USA, #13901, 1:1000), and anti-Histone H3 (Cell Signaling Technology, USA, #4499, 1:1000). After washing, membranes were incubated with Goat anti–rabbit HRP antibody (Abclonal, China, #AS014, 1:10000) or Goat anti–mouse HRP antibody (Abclonal, China, #AS003, 1:10000) at room temperature for 1 h. Chemiluminescence detection was performed with an ECL kit (FDbio, China, #FD8000), and the band intensity was semi-quantified using ImageJ.

### 2.16 Mitochondrial oxygen consumption rate (OCR) and mitochondrial extracellular acidification rate (ECAR) analysis

The mitochondrial OCR and ECAR of arterial PVAT-derived SVF adipocytes (from saline- and CL-treated atherosclerotic mice) were measured using a Seahorse Bioscience XF96 extracellular flux analyzer through classical real-time and live-cell analyses according to the manufacturer's protocol (Agilent Technologies). For OCR measurement, the differentiation medium was replaced with XF assay medium containing 1 mM pyruvate, 2 mM glutamine and 10 mM glucose at pH 7.4. After baseline measurement, the oxidative phosphorylation inhibitor oligomycin (1 μM), FCCP (1.5 μM) and rotenone/antimycin (1 μM/5 μM) were added for the measurement of mitochondrial ATP production, maximal respiration, spare respiration capacity, respectively. For ECAR measurement, the differentiation medium was replaced with XF assay medium containing 2 mM glutamine at pH 7.4. After baseline measurement, glucose (10 mM), oligomycin (1 mM), and glycolytic inhibitor 2-DG (50 mM) were injected into each well at indicated time points according to the manufacturer's protocol for the measurement of cellular glycolytic function (Agilent Technologies).

### 2.17 Assessment of aortic atherosclerotic lesion area

For atherosclerotic lesions assessment, the whole aorta was dissected and opened along the ventral axis. Aortas were then stained with Oil Red O solution (Beyotime Biotechnology, China, #C0157S). Images of the whole aorta were captured with a digital camera (Nikon SMZ1500, Japan) and were analyzed using ImageJ software. The 5 μm-thick frozen sections of aortic root were analyzed by Oil Red O staining, H&E staining (Beyotime Biotechnology, China, #C0105S), Movat staining (Powerful Biology), and Masson staining (Beyotime Biotechnology, China, #C0189S) according to manufacturer's instructions. Quantitative analysis of lesions was performed using ImageJ software with n = 6 or more.

### 2.18 Immunofluorescence (IF) analysis

The 5 μm-thick frozen sections were collected and air-dried for 30 min at room temperature followed by washing in PBS. And then all slides were permeabilized using 0.25% Triton X-100 in PBS for 10 min. After washing 3 times with PBS, slides were blocked in blocking buffer (5% horse serum and 1% bovine serum albumin in PBS) for 1 h at room temperature. All slides were then incubated with anti-CD68 (Bio-Rad Laboratories, Inc., USA, #MCA341GA, 1:250) or anti-MMP9 (Abcam, #ab283575, 1:100) primary antibody in blocking buffer overnight at 4°C. After washing, the sections were incubated for 1 h at room temperature with either Goat anti-Rat IgG (H + L) Alexa Fluor 594 (Invitrogen, USA, #A-11007, 1:500) or Goat anti-Rabbit IgG (H + L) Alexa Fluor 594 (Invitrogen, USA, #A-11012, 1:500), together with 4′,6-diamidino-2-phenylindole (DAPI) (Solarbio Life Sciences, China, #D8200, 1:1000) for nuclear counterstaining. All slides were mounted with Antifade Mounting Medium (Thermo Fisher, USA, #S36937) and imaged by a microscope (Olympus, FV3000). The positive fluorescence intensity of corresponding antibodies staining was determined via Image J software with n = 6 or more.

### 2.19 Plasma lipid profile analysis

The whole blood samples were harvested from the left ventricle and collected in anticoagulant tubes. After centrifugation, plasma levels of total cholesterol (TC), low-density lipoprotein-cholesterol (LDL-C) and high-density lipoprotein-cholesterol (HDL-C) were determined by the Center for Drug Safety Evaluation and Research of Zhejiang University.

### 2.20 Blood pressure measurement

Blood pressure was measured via a noninvasive tail cuff apparatus coupled to a PC-based data acquisition system (RTBP1007; Kent Scientific, Litchfield, Connecticut, USA) between 1 p.m. and 5 p.m. Blood pressure values were derived from an average of three measurements per mouse at each time point.

### 2.21 Transcription factor predication analysis

The potential *GPX4* transcription factors (TFs) and the corresponding TFs binding motif were predicated via the JASPAR Database (https://jaspar.genereg.net/).

### 2.22 CUT&Tag analysis

CUT&Tag was performed using the Hyperactive Universal CUT&Tag Assay Kit for Illumina (Vazyme Biotech, TD904) according to the manufacturer's instructions. Nuclei were isolated from 5 × 10^4^ adipocytes per sample and lightly cross-linked with 0.1% formaldehyde for 2 min at room temperature, followed by quenching with 75 mM glycine. Nuclei were then immobilized onto ConA Beads Pro and incubated overnight at 4°C with 1 μL of primary antibody (Normal Mouse IgG, Sigma-Aldrich, 12-371B; Anti-Flag, Cell Signaling Technology, 8146) in 50 μL antibody buffer. After washing, beads were incubated with 1 μL secondary antibody (Goat Anti-Mouse H&L, Vazyme Biotech, ab208-01) in 50 μL Dig-wash buffer for 1 h at room temperature. Following three washes, beads were incubated with 2 μL pA/G-Tnp Pro in 98 μL Dig-300 Buffer for 1 h at room temperature, washed again, and then incubated with 10 μL of 1× TTBL in Dig-300 Buffer at 37°C for 60 min. Next, 0.5 pg DNA spike-in and 2 μL 10% SDS were added, and samples were heated at 55°C for 10 min. Supernatants were collected, incubated with DNA Extract Beads Pro for 20 min at room temperature, washed twice with B&W buffer, and resuspended in 15 μL ddH_2_O. Libraries were amplified and sequenced on the NovaSeq XP platform (150-bp paired-end reads). Quality control was performed with an Agilent 2100 Bioanalyzer, and data were analyzed using the cut_tag_tool from Vazyme (http://cloud.vazyme.com:83/).

### 2.23 Luciferase reporter gene assays

The human C/EBPβ plasmids were purchased from WZ Biosciences *Inc.* (Shandong, China). The *GPX4* promoter plasmid (−2000 to 0) was purchased from Miaoling Plasmid (#P90845). The five single mutant plasmid constructs were generated through site-directed mutagenesis. For luciferase report assay, 293T cells were co-transfected with 1 μg of GPX4 plasmid (WT or Mutant 1-5), 1 μg of empty vector or C/EBPβ and 0.2 μg pRL-TK (Promega, E2241). Cells were cultured for 48 h and detected using the Dual Luciferase Reporter Gene Assay Kit (YEASEN, 11402ES60) following the manufacturer's protocol. The luciferase activity was assayed using a microplate reader (VICTOR Nivo, PerkinElmer).

### 2.24 Subcellular fractionation isolation

Arterial PVAT-derived SVF adipocytes were treated by vehicle (DMSO) or CL (1 μM) for two days. After that, cytosolic and nuclear fractions of these cells were isolated by differential centrifugation. Briefly, cells were washed with ice-cold PBS and homogenized in KPBS solution (potassium-phosphate buffered saline, containing 136 mM KCl, 10 mM KH_2_P0_4_, pH 7.3, dilution in PBS buffer) with EDTA-free proteases inhibitor cocktail by using a teflon-glass hand homogenizer. The homogenized samples were spun at 600 ×g for 10 min. The lysate was collected and further centrifuged at 16000 ×g for 10 min. The supernatant served as cytosolic fraction. The pellet nuclei were resuspended and vortexed in isolation solution (20 mM HEPES (pH 7.4), 10 mM KCL, 2 mM MgCL_2_, 0.5% NP40, 0.5 M NaCl, dilution in distilled water) with EDTA-free proteases inhibitor cocktail and centrifuged at 2500 ×g for 3 min. After three time washed by lysis solution, the pellet served as nuclear fraction, and the pellet then resuspend in cold lysis buffer for nuclear fraction lysates. The Nuclear/Cytoplasmic fraction ratio is analyzed by Image J software.

### 2.25 GEO dataset analysis

The raw data of GSE28440 was downloaded from the public dataset in the Gene expression omnibus (GEO) database contained SFD-fed or HFD-fed C57BL/6J mice arterial PVAT RNA sequencing samples (three biological replicates). All raw data were quality-controlled, background-corrected, normalized, log-transformed, and processed for batch effects removal via the R packages “limma”. Data were normalized and screened differential expression genes (DEGs) with false discovery rate (FDR) < 0.05 and |log2FC| ≥1. DEGs were uploaded to the DAVID website (https://david.ncifcrf.gov) for GO and KEGG signaling pathway enrichment annotations.

### 2.26 Quantification and statistical analysis

All results are presented as the mean ± SD and were analyzed using GraphPad Prism 10. Before statistical comparisons among the experimental data were performed, the normality of the data distribution was checked by the Shapiro‒Wilk test. For data with a normal distribution, unpaired *t*-test (two tailed) was used to compare two groups, one-way ANOVA followed by Tukey's multiple comparisons test was used to compare the means among multiple groups, one-way ANOVA followed by Dunnett's multiple comparisons test was used for multiple comparisons against a control group, and two-way ANOVA analysis followed by a Bonferroni multiple comparison test was used for grouped analyses. For non-normally distributed data, Mann-Whitney test was applied for comparisons between two groups and Kruskal-Wallis test with Dunn's multiple comparisons test were applied for comparisons among multiple groups. For correlation analyses between two pairs of columns, values were assessed using a two-tailed Pearson correlation analysis. Statistical analyses were performed in at least three or more independent values. *P* value < 0.05 was considered statistically significant.

## 3 Results

### 3.1 PVAT ferroptosis in established atherosclerotic plaque

**Fig. 1:**
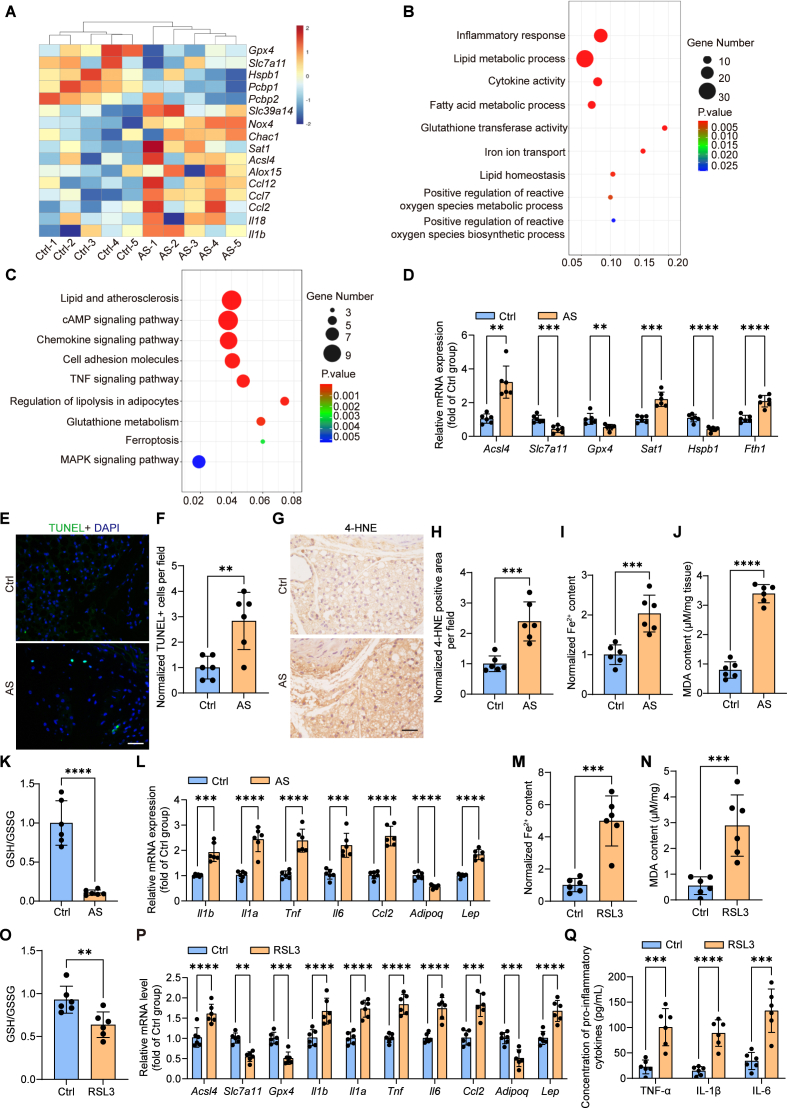
Ferroptosis in established atherosclerotic PVAT. (A) Heatmap representing the genes differentially expressed between control PVAT and atherosclerotic PVAT, as determined by RNA-sequencing analysis, in which data were sorted as log (fold change) ≥ 1.5 or ≤ 1.5 and p < 0.05 (n = 5 per group). (B) Dot plot of GO items enriched in differentially expressed genes in control PVAT and atherosclerotic PVAT based on RNA-sequencing data. (C) Dot plot of KEGG items enriched in differentially expressed genes in control PVAT and atherosclerotic PVAT based on RNA-sequencing data. (D) mRNA levels of genes closely associated with ferroptosis in PVAT from control and atherosclerotic groups, n = 6. (E-F) TUNEL staining in PVAT from control and atherosclerotic groups. Representative images are shown in (E) with quantification of normalized TUNEL positive cells in (F). Scale bar represents 50 μm, n = 6. (G-H) Immunohistochemical analysis of 4-HNE in PVAT from control and atherosclerotic groups. Representative images are shown in (G) with quantification of normalized 4-HNE-positive area in (H). Scale bar represents 50 μm, n = 6. (I–K) Measurement of normalized Fe^2+^ content (I), MDA (J), and GSH/GSSG ratio (K) in PVAT homogenates from control and atherosclerotic groups. (L) mRNA levels of genes associated with PVAT dysfunction in control and atherosclerotic groups, n = 6. (M − O) Measurement of normalized Fe^2+^ content (M), MDA (N), and GSH/GSSG ratio (O) in PVAT explants treated by vehicle (DMSO) or RSL3 (5 μM) for 24 h, n = 6. (P) mRNA levels of genes associated with ferroptosis and PVAT dysfunction in in PVAT explants treated by vehicle (DMSO) or RSL3 (5 μM) for 24 h, n = 6. (Q) ELISA analysis of TNF-α, IL-1β and IL-6 levels in the conditioned medium of PVAT explants treated by vehicle (DMSO) or RSL3 (5 μM) for 24 h, n = 6. The quantification data are from at least 6 independent biological samples per group and are shown as the mean ± SD. Differences in (D, F, H-Q) were analyzed by unpaired *t*-test. **P < 0.01, ***P < 0.001, ****P < 0.0001. Ctrl, Control; AS, Atherosclerosis.

To investigate ferroptosis in perivascular adipose tissue (PVAT) in established atherosclerosis, we performed RNA-seq analysis on PVAT samples from two groups: control *Apoe*^−/−^ mice before high-fat diet (HFD) feeding and *Apoe*^−/−^ mice with established atherosclerosis after 16 weeks of HFD. Transcriptomic analysis revealed that, compared to the control group, ferroptosis-suppressive genes, including *Gpx4*, were significantly downregulated in the atherosclerotic group. Conversely, ferroptosis-promoting genes, including *Acsl4*, and inflammatory cytokine genes, including *Il1b*, were markedly upregulated in the atherosclerotic group (Fig. 1A). Gene Ontology (GO) enrichment analysis showed that differentially expressed genes were enriched in biological processes associated with ferroptosis, such as inflammation, lipid metabolic process, glutathione transferase activity, iron ion transport, and oxidative stress (Fig. 1B). Pathway analysis further demonstrated associations with lipid peroxidation, atherosclerosis, cAMP pathway, chemokine signaling pathways, and ferroptosis (Fig. 1C, Fig. S1). Notably, an independent RNA-seq (GSE28440) from PVAT of C57BL/6 mice also indicated that high fat-diet (HFD) promotes ferroptosis. In this cohort, differential expressed genes (DEGs) were enriched in process such as inflammation, oxidative stress, ROS synthesis and intrinsic apoptosis, while DEG-associated pathways were closely tied to lipids, atherosclerosis, the TNF and chemokine signaling pathway, peroxisomes, ROS and ferroptosis (Fig. S2). These findings collectively establish PVAT ferroptosis as a novel pathological mechanism in HFD-induced atherosclerosis. To validate these observations, we confirmed key ferroptosis-related genes through qPCR. Results demonstrated significantly increased mRNA levels of pro-ferroptosis genes (e.g., *Acsl4*) and reduced anti-ferroptosis genes (e.g., *Gpx4*) in atherosclerotic PVAT (Fig. 1D). In addition, TUNEL staining and histological staining of 4-HNE further confirmed increased cell death and lipid peroxidation markers in atherosclerotic PVAT samples (Fig. 1E–H). Biochemical assays confirmed elevated ferrous ion (Fe^2+^) concentrations, MDA content, and decreased GSH/GSSG ration in diseased PVAT compared to controls (Fig. 1I–K). These data collectively confirm the cell death accompanied by lipid peroxidation and iron accumulation, exhibiting characteristic features of ferroptosis in atherosclerotic PVAT. As ferroptosis in adipose tissue may disrupt mitochondrial function and promote adipose dysfunction through lipid peroxidation, oxidative stress, and proinflammatory cytokine release [39], we further determined inflammatory gene expression in PVAT. qPCR results showed significantly elevated proinflammatory cytokine mRNA levels in atherosclerotic PVAT, consistent with RNA-seq findings (Fig. 1L). Correlation analysis between representative ferroptosis markers and adipose dysfunction indicators revealed statistically significant associations (Fig. S3), suggesting that ferroptosis is a key pathological driver of PVAT dysfunction in atherosclerosis. To directly test this hypothesis, we isolated PVAT and treated explants *ex vivo* with the ferroptosis inducer RSL3 (5 μM). RSL3 treatment significantly enhanced key markers of ferroptosis, including elevated intracellular Fe^2+^ and MDA levels, as well as a markedly reduced GSH/GSSG ratio (Fig. 1M–O). Concurrently, qPCR analysis revealed upregulated mRNA expression of pro-ferroptotic genes and downregulated expression of anti-ferroptotic genes. Moreover, we observed significantly increased mRNA levels of proinflammatory cytokines and dysregulated adipokine expression in RSL3-treated PVAT (Fig. 1P). Consistent with these findings, ELISA analysis of conditioned media showed substantially higher secretion of proinflammatory cytokines from RSL3-treated PVAT compared to vehicle (DMSO)-treated controls (Fig. 1Q). These findings highlight ferroptosis as a critical pathological mechanism contributing to PVAT dysfunction and atherosclerosis.

### 3.2 CL-316243 ameliorates arterial PVAT ferroptosis

**Fig. 2:**
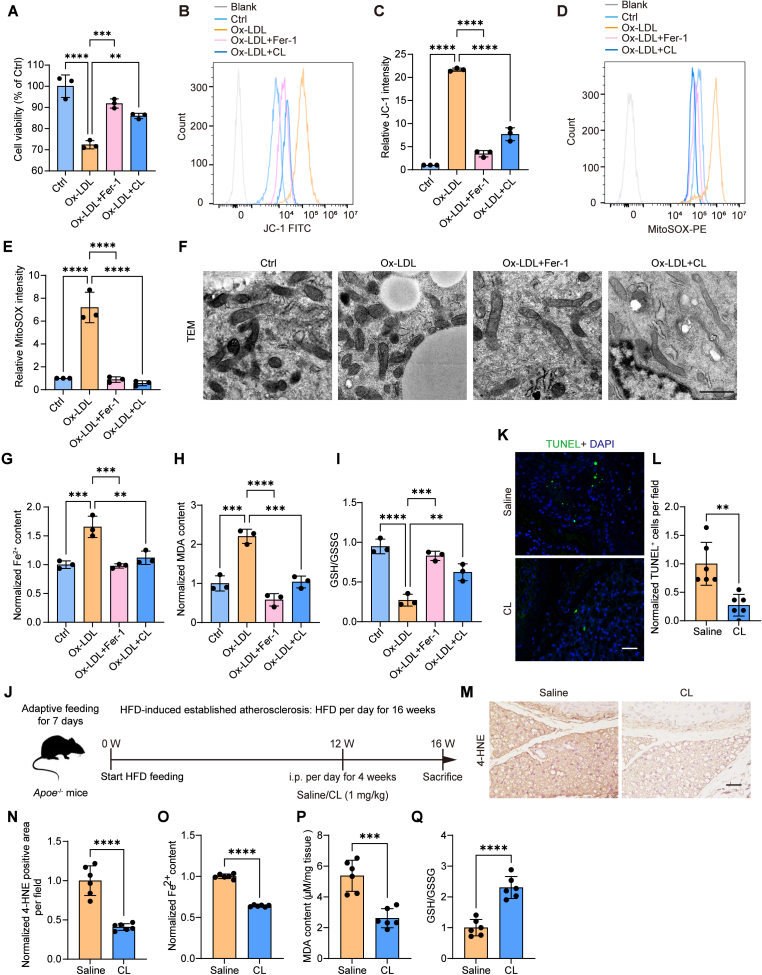
CL suppresses ferroptosis *in vitro* and *in vivo*. (A) Cell viability of arterial PVAT-derived adipocytes after treatment with vehicle control (Ctrl), Ox-LDL (150 μg/mL), Ox-LDL (150 μg/mL) plus the ferroptosis inhibitor ferrostatin-1 (Fer-1, 5 μM), or Ox-LDL (150 μg/mL) plus CL (1 μM). Cells were first pretreated with PBS (as vehicle control) or Ox-LDL for 24 h, followed by a second 24-h treatment with Ox-LDL in combination with either vehicle (DMSO), Fer-1, or CL. n = 3. (B–C) Analysis of mitochondrial membrane potential in adipocytes under the treatment conditions described in (A), as assessed by flow cytometry. Representative histograms of JC-1 and FITC are shown in (B), and the normalized JC-1 and FITC median fluorescence intensity (MFI) is shown in (C) (n = 3). (D-E) Analysis of mitochondrial ROS in adipocytes under the treatment conditions described in (A), as assessed by flow cytometry. Representative histograms of MitoSOX and PE are shown in (D), and the normalized MitoSOX and PE MFI is shown in (E) (n = 3). (F) Representative transmission electron microscopy images of mitochondrial morphology in adipocytes treated as described in (A). Scale bar, 1 μm. (G-I) Determination of Fe^2+^ content (G), normalized MDA content (H), and ratio of GSH/GSSG (I) in adipocytes under the treatment conditions described in (A) (n = 3). (J) Schematic diagram showing the *in vivo* administration of CL. *Apoe*^*−/−*^ mice were fed with high-fat diet (HFD) for 16 weeks to induce established atherosclerotic plaque. Saline or CL-316243 (CL; 1 mg/kg/day) was administrated from week 12 of HFD feeding for consecutive 4 weeks. (K-L) TUNEL staining in PVAT from saline or CL treated mice. Representative images are shown in (K) with quantification of normalized TUNEL positive cells in (L). Scale bar represents 50 μm, n = 6. (M − N) Immunohistochemical analysis of 4-HNE in PVAT from saline or CL treated mice. Representative images are shown in (M) with quantification of normalized 4-HNE-positive area in (N). Scale bar represents 50 μm, n = 6. (O-Q) Measurement of Fe^2+^ content (O), MDA (P), and GSH/GSSG ratio (Q) in PVAT homogenates from saline or CL treated mice. The quantification data are shown as the mean ± SD. Differences were analyzed by one-way ANOVA with post hoc Tukey's multiple comparisons test (A, C, E, G-I) or unpaired *t*-test (L, N-Q). **P < 0.01, ***P < 0.001, ****P < 0.0001. Ctrl, Control; CL, CL-316243; Ox-LDL, Oxidized low-density lipoprotein; Fer-1, ferrostatin-1.

Our results described above suggest that HFD promotes ferroptosis in PVAT. Pericoronary adipose tissue has been identified as a storage and supply site for Ox-LDL in human coronary plaques [40]. Moreover, Ox-LDL is known to drive ferroptosis in endothelial cells and macrophages [16,41]. These findings suggest that Ox-LDL may play a central role in PVAT ferroptosis and atherosclerosis. To validate this hypothesis, we treated arterial PVAT-derived SVF adipocytes with Ox-LDL (150 μg/mL). After stimulation, cells exhibited increased cellular injury, reduced mitochondrial membrane potential, increased oxidative stress, shrunken mitochondrial morphology, elevated Fe^2+^ level, MDA content, and decreased GSH/GSSG ratio. In addition, these ferroptosis-associated features were reversed by ferroptosis inhibitor Ferrostatin-1 (Fer-1, 5 μM), confirming that Ox-LDL triggers PVAT ferroptosis (Fig. 2A–I). Ox-LDL induces elevated reactive oxygen species (ROS) production, which may underlie PVAT ferroptosis and subsequent dysfunction. To further elucidate the role of ROS in this process, we treated adipocytes with a ROS scavenger N-acetylcysteine (NAC, 5 μM). The results showed that ROS scavenging significantly reversed Ox-LDL induced ferroptotic markers: it attenuated the elevated levels of Fe^2+^ and MDA, and restored the reduced GSH/GSSG ratio (Fig. S4A–C). Moreover, the ROS scavenger suppressed Ox-LDL-driven upregulation of pro-ferroptotic genes and prevented the downregulation of anti-ferroptotic genes (Fig. S4D–E). Importantly, it also ameliorated Ox-LDL-induced increases in proinflammatory gene expression and normalized dysregulated adipokine mRNA levels (Fig. S4F–I). In addition, ELISA analysis of conditioned media showed substantially lower secretion of proinflammatory cytokines from NAC-treated adipocytes compared to Ox-LDL-treated group (Fig. S4J–L). Collectively, these findings indicate that Ox-LDL triggered ROS generation is a critical driver of ferroptosis and functional impairment in PVAT.

Meanwhile, to examine the effect of CL on inhibiting Ox-LDL-induced adipocyte ferroptosis, we also evaluated the effect of CL on cell viability, mitochondrial membrane potential and morphology, the redox system, and Fe^2+^ content in Ox-LDL-treated cells. Compared to cells treated with Ox-LDL, CL (1 μM) treatment significantly suppressed ferroptosis-related cell damage, restoring mitochondrial membrane potential and morphology, lowing Fe^2+^ content, and reducing lipid peroxidation (Fig. 2A–I). These findings demonstrate that CL exerts ferroptosis-inhibitory effects *in vitro*. To further investigate whether CL suppresses arterial PVAT ferroptosis *in vivo*, CL (1 mg/kg) or vehicle (saline) were intraperitoneally injected for 4 continuous weeks from week 12 of high-fat diet (HFD) feeding (Fig. 2J). Next, we determined ferroptosis-associated markers in PVAT from these mice. CL treatment reduced TUNEL positive cells, indicating that CL inhibited adipocyte death in arterial PVAT (Fig. 2K and L). To evaluate the effect of CL on lipid peroxidation *in vivo*, we performed IHC staining for 4-HNE. CL administration reduced 4-HNE positive area (Fig. 2M and N). Corresponding to this, biochemical assays confirmed lower Fe^2+^ and MDA content, higher GSH/GSSG ratio in PVAT homogenate from CL treated mice (Fig. 2O–Q). Collectively, these results suggest that CL effectively suppresses PVAT ferroptosis.

### 3.3 CL-316243 inhibits arterial PVAT dysfunction in established atherosclerotic mice

**Fig. 3:**
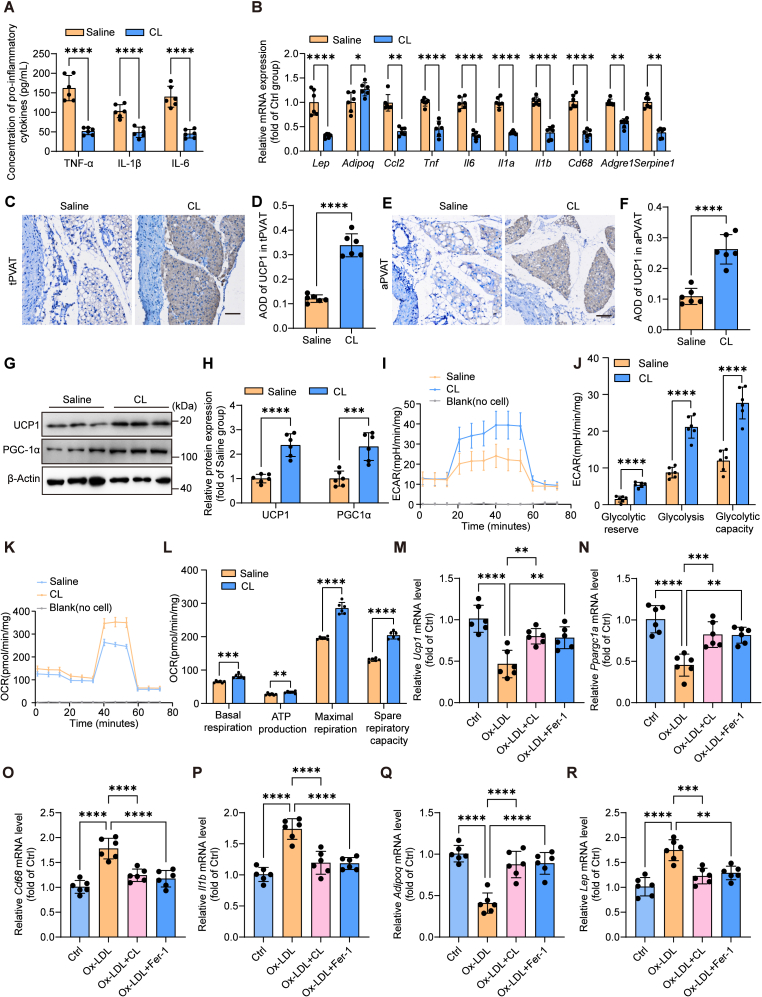
CL-316243 improves arterial PVAT function in atheroscler**otic mice.** (A) ELISA analysis of TNF-α, IL-1β and IL-6 levels in the conditioned medium of arterial PVAT from saline- or CL-treated mice. The quantification data are from at least 6 independent biological samples per group. (B) The relative mRNA level of vasoactive factors and genes involved in inflammation in arterial PVAT from saline- or CL-treated mice, n = 6. (C–F) Histological analysis of UCP1 in thoracic aortic PVAT (tPVAT) and abdominal aortic PVAT (aPVAT) from saline- or CL-treated *Apoe*^−/−^ mice. Representative IHC images of UCP1 are shown in (C, E) with quantification of the average optical density (AOD) of UCP1 in tPVAT and aPVAT in (D, F). Scale bar, 50 μm. n = 6. (G-H) Western blotting analysis of UCP1 and PGC-1α in arterial PVAT of saline- or CL-treated mice. Representative blots are shown in (G) with normalized quantification in (H). (I-L) Cellular and mitochondrial energy metabolism in isolated arterial PVAT-derived adipocytes from saline or CL treated *Apoe*^−/−^ mice. The extracellular acidification rate (ECAR) and oxygen consumption rate (OCR) of adipocytes are shown in (I-J) and (K-L), respectively. (n = 6). (M-R) The relative mRNA level of *Ucp1*, *Ppargc1a*, *Cd68*, *Il1b*, *Adipoq*, and *Lep* from PVAT explants as indicated. PVAT explants were first pretreated with PBS (as vehicle control) or Ox-LDL (75 μg/mL) for 24 h, followed by a second 24-h treatment with Ox-LDL in combination with either vehicle (DMSO), Fer-1 (5 μM), or CL (1 μM). n = 6. The data are presented as the mean ± SD. Differences were assessed using unpaired *t*-test (A-B, D, F, H, J, L) or one-way ANOVA with post hoc Tukey's multiple comparisons test (M-R). *P < 0.05, **P < 0.01, ***P < 0.001, ****P < 0.0001. Ctrl, Control; CL, CL-316243.

Increased oxidative stress and inflammation in adipose tissue leads to PVAT dysfunction. This dysfunction promotes macrophage infiltration and proinflammatory cytokine release, which may trigger vulnerable plaques [13,42]. Thus, we next determined the concentration of proinflammatory cytokines in the conditioned medium of arterial PVAT isolated from saline- or CL-treated mice. ELISA analysis revealed significantly lower levels of proinflammatory cytokines in the conditioned media of PVAT from CL-treated mice compared to saline-treated controls (Fig. 3A). Consistently, the mRNA levels of genes involved in vessel constriction, inflammation, and macrophage infiltration in arterial PVAT from CL-treated mice was significantly reduced (Fig. 3B), indicating that CL suppresses inflammatory cell infiltration and arterial PVAT inflammation. These results suggest that CL treatment prevents arterial PVAT dysfunction and reduces releasement of arterial PVAT-derived vasoactive factors in established atherosclerotic mice.

To further explore the role of CL in ameliorating PVAT dysfunction induced by lipid oxidative stress, we therefore determined UCP1 protein expression patterns and mitochondrial function in PVAT from these mice. IHC staining of UCP1 showed that CL treatment increased the average optical density of UCP1 in thoracic PVAT and abdominal aortic PVAT (Fig. 3C–F). Western blotting analysis confirmed that CL treatment upregulated UCP1 and peroxisome proliferator-activated receptor gamma coactivator-1 (PGC-1α) (Fig. 3G and H), which are critical in nonshivering thermogenesis, mitochondrial metabolism, mitochondrial DNA replication regulation, mitochondrial gene expression, and mitochondrial function in brown adipose tissue [43]. Next, glycolytic and mitochondrial oxidative phosphorylation function were further examined by measuring the extracellular acidification rate (ECAR) and mitochondrial oxygen consumption rate (OCR). CL treatment enhanced both glycolytic function and mitochondrial oxidative phosphorylation in arterial PVAT adipocytes. Specifically, CL increased glycolysis, maximal glycolytic capacity, basal oxygen consumption, mitochondria-linked ATP production, maximal mitochondrial respiration, and spare respiratory capacity compared to saline-treated controls (Fig. 3I–L). We next validated the protective effects of CL in *ex vivo* PVAT explants. Ox-LDL (75 μg/mL) treatment markedly suppressed the mRNA expression of thermogenic markers *Ucp1* and *Ppargc1α* in PVAT (Fig. 3M and N); however, co-administration of CL (1 μM) significantly attenuated this downregulation. Notably, the efficacy of CL closely resembled that of the ferroptosis inhibitor Fer-1 (5 μM). In parallel, CL also reversed Ox-LDL-induced dysregulation of proinflammatory cytokines and adipokine transcripts in PVAT, with effects similar to those observed with ferroptosis inhibition (Fig. 3O–R). Collectively, these findings indicate that CL alleviates Ox-LDL induced PVAT dysfunction.

### 3.4 CL-316243 facilitates stable atherosclerotic plaque phenotypes

**Fig. 4:**
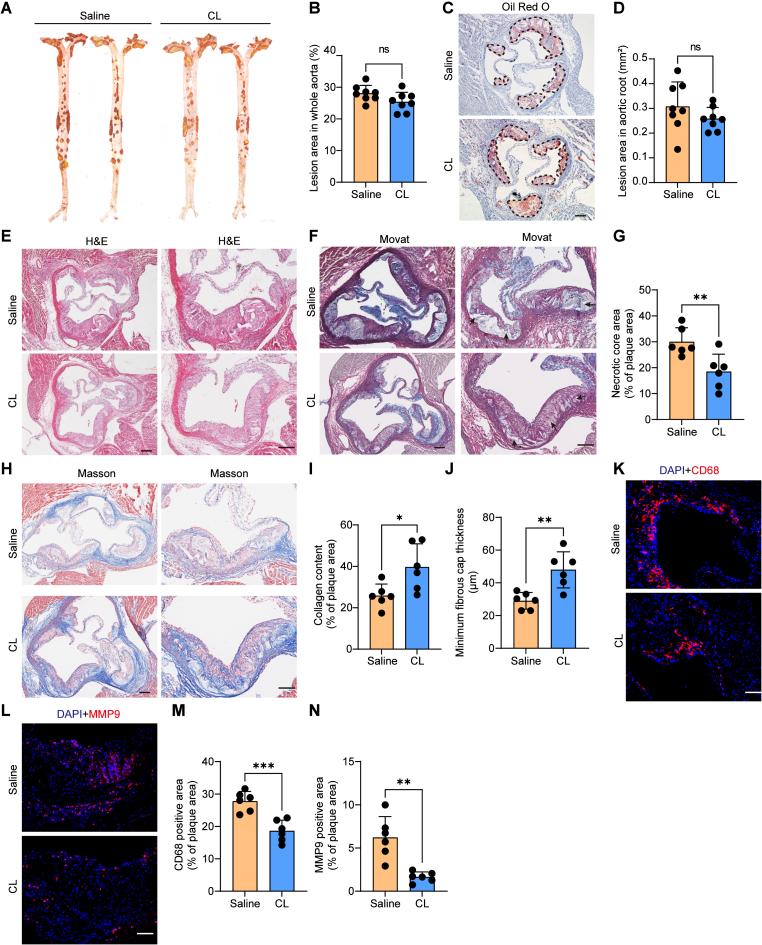
CL-316243 facilitates stable plaque phenotypes in mice with established athero**sclerosis.** (A-B) Histological analysis of atherosclerotic plaques in the whole aortas of saline or CL treated mice. Representative Oil red O-stained images are shown in (A) and quantification of the percentage of lesion area to surface area of whole aorta (n = 8) is shown in (B). (C-D) Histological analysis of atherosclerotic plaques in aortic roots from saline or CL treated mice. Representative images of Oil red O-stained aortic roots are shown in (C) with quantification of lesion area in (D) (n = 8). Scale bar, 200 μm. (E-F) Representative images of H&E and Movat staining of aortic roots from saline or CL treated mice. The necrotic cores are indicated by arrows. Scale bar, 200 μm at low magnification and 50 μm at high magnification. (G) Quantification of the percentage of necrotic core area to plaque area in aortic root according to Movat staining (n = 6). (H) Masson staining of aortic roots from saline or CL treated mice. Scale bar, 200 μm at low magnification and 50 μm at high magnification. (I) Quantification of the content of collagen in plaque (n = 6). (J) Quantification of the minimum fibrous cap thickness in plaque (n = 6). (K, M) Immunofluorescence staining analysis of CD68 in aortic roots from saline or CL treated mice. Representative images are shown in (K) with quantification of the percentages of CD68 positive area in the aortic root lesion in (M) (n = 6). Scale bar, 50 μm. (L, N) Immunofluorescence staining analysis of MMP9 in aortic roots from saline or CL treated mice. Representative images are shown in (L) with quantification of the percentages of MMP9 positive area in the aortic root lesion in (N) (n = 6). Scale bar, 50 μm. The data are presented as the mean ± SD. Differences were assessed using unpaired *t*-test. *P < 0.05, **P < 0.01, ***P < 0.001. ns, not significant. CL, CL-316243.

Next, we investigate the effects of CL on established atherosclerosis. Following 16 weeks of HFD feeding, atherosclerotic lesions were evaluated in the whole aorta and aortic root by histological staining. Interestingly, there is no significant reduction of atherosclerotic lesion area in CL (1 mg/kg) treated mice compared with the saline group, as evidenced by histological analysis of aortas and aortic roots (Fig. 4A–D). However, CL-treated mice showed smaller necrotic cores by hematoxylin-eosin (H&E) and Movat staining (Fig. 4E–G) and increased collagen and thicker fibrous caps by Masson staining (Fig. 4H–J), contributes to stable plaque phenotypes. To elucidate the underlying mechanisms, we further explored cellular components within atherosclerotic lesions. Macrophage content was determined by calculating the percentage of the total plaque area positively stained for CD68. As shown in (Fig. 4K–M), the percentage of plaque area that was CD68-positive was significantly reduced in CL-treated mice after 16 weeks of HFD, indicating a decrease in macrophage infiltration. Furthermore, the expression of MMP9 in the aortic roots in CL-treated mice was also significantly reduced (Fig. 4L–N). Notably, these plaque stability indicators correlated significantly with MDA content and the GSH/GSSG ratio in corresponding PVAT tissue (Fig. S5). Notably, CL treatment did not significantly alter plasma total cholesterol or low-density lipoprotein (LDL) cholesterol levels in these mice (Fig. S6A–C), consistent with previous reports [44,45]. In addition, CL administration was associated with a modest reduction in systolic blood pressure, while diastolic blood pressure and heart rate remained unchanged (Fig. S6D–F), indicating that the observed improvements in stable plaque phenotypes were not accompanied by detectable changes in systemic lipid profile or hemodynamic parameters at the dose tested. This suggests a direct relationship between PVAT ferroptosis and plaque instability, highlighting CL's potential to modulate atherosclerotic plaque vulnerability through PVAT-targeted mechanisms.

### 3.5 CL-316243 protects arterial PVAT from ferroptosis by upregulating GPX4 expression via the cAMP-PKA-C/EBPβ pathway

**Fig. 5:**
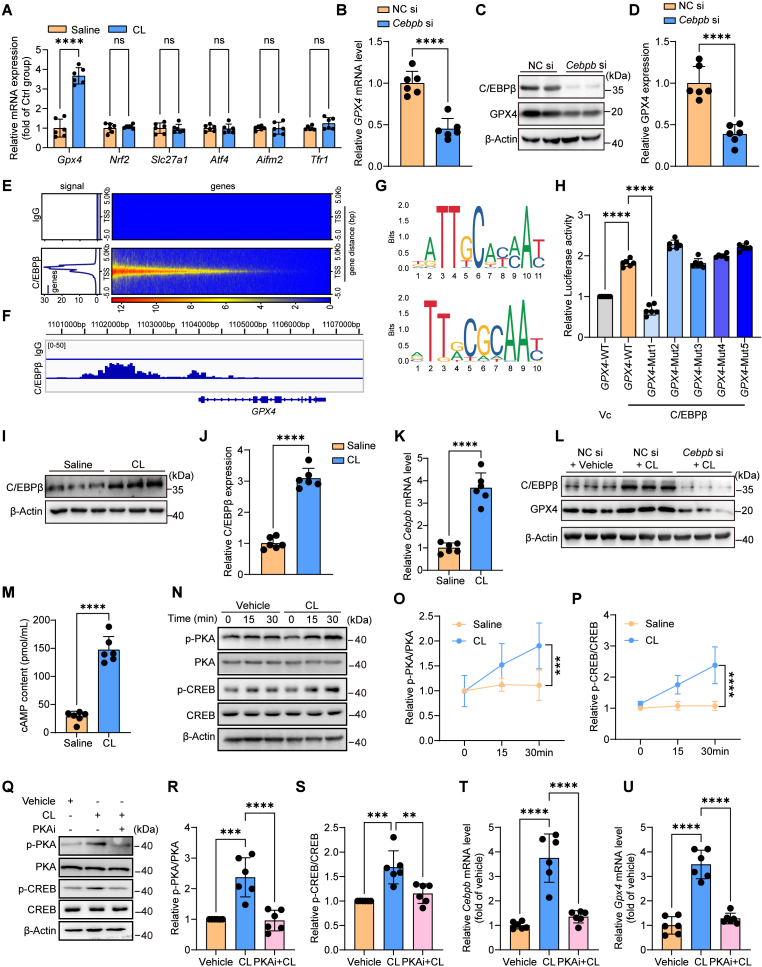
**CL-316243 promotes expression of the ferroptosis suppressor GPX4 in arterial PVAT via cAMP-PKA-C/EBPβ axis.** (A) Relative mRNA expression levels of *Gpx4*, *Nrf2*, *Slc27a1*, *Atf4*, *Aifm2*, and *Tfr1* in arterial PVAT from saline- or CL-treated mice (n = 6). (B) Relative *Gpx4* mRNA levels in arterial PVAT-derived adipocytes transfected with NC si or *Cebpb* si. (C-D) WB analysis of GPX4 protein expression in adipocytes transfected with NC si or *Cebpb* si. Representative blots are shown in (C), and the normalized quantification is shown in (D). (E-F) Enrichment and genome browser view of negative controls (IgG) and C/EBPβ at the *GPX4* promoter in 3T3-L1 adipocytes. (G) Predicted sites of the putative C/EBPβ binding motif by the JASPAR database. (H) Luciferase activity in 293T cells transfected with *GPX4*-WT or five *GPX4*-Muts (*GPX4*-Mut1-5) promotor plasmid in the presence of vector or C/EBPβ plasmid for 48 h*.* The relevant luciferase activity values were based on the Renilla control (n = 6). (I-J) WB analysis of C/EBPβ protein expression in arterial PVAT from saline or CL treated *Apoe*^*−/−*^ mice. Representative blots are shown in (I), and the normalized quantification is shown in (J) (n = 6). (K) Normalized *Cebpb* mRNA levels in arterial PVAT of saline or CL treated mice (n = 6). (L) Representative blots of C/EBPβ and GPX4 in control and *Cebpb* knockdown adipocytes with or without CL treatment. (M) ELISA analysis of cyclic adenosine monophosphate (cAMP) in adipocytes after vehicle or 1 μM CL treatment for 6 h (n = 6). (N–P) WB analysis of *p*-PKA (Thr197) and *p*-CREB (Ser133) in adipocytes treated with vehicle (DMSO) or CL (1 μM) for 0, 15, and 30min. Representative blots of *p*-PKA, PKA, *p*-CREB, and CREB are shown in (N), and the normalized quantification is shown in (O–P). n = 6. (Q-S) WB analysis of *p*-PKA (Thr197) and *p*-CREB (Ser133). Adipocytes were pretreated with vehicle (DMSO) or PKA inhibitor (20 μM) for 1 h followed by vehicle (DMSO) or CL (1 μM) treatment for 30min. Representative blots of *p*-PKA, PKA, *p*-CREB, and CREB are shown in (Q), and the normalized quantification is shown in (R–S). n = 6. (T-U) Relative *Cebpb* (T) and *Gpx4* (U) mRNA levels (n = 6). Adipocytes were pretreated with vehicle (DMSO) or PKA inhibitor (20 μM) for 1 h followed by vehicle (DMSO) or CL (1 μM) treatment for 6 h. Quantification data are shown as the mean ± SD. Differences were assessed using unpaired *t*-test (A, B, D, J, K, M), one-way ANOVA with post hoc Tukey's multiple comparisons test (H, R–U), and two-way ANOVA followed by Bonferroni multiple comparison test (O–P). **P < 0.01; ***P < 0.001; ****P < 0.0001. NC, negative control; Mut, Mutation; CL, CL-316243; PKAi, PKA inhibitor.

To elucidate the molecular mechanism by which CL inhibits PVAT ferroptosis, we analyzed key ferroptosis-associated genes, including *Gpx4*, nuclear factor erythroid-related factor 2 (*Nrf2*), solute carrier family 7 member 11 (*Slc7a11*), transcription factor 4 (*Atf4*), ferroptosis suppressor protein 1 (*Aifm2*), and transferrin receptor protein 1 (*Tfr1*). Among the ferroptosis-related genes examined, *Gpx4* exhibited the most pronounced and consistent regulation by CL (Fig. 5A). We therefore selected *Gpx4* for further mechanistic investigation. Western blotting (Fig. S7A–B) and IHC staining (Fig. S7C–F) subsequently confirmed that CL upregulates GPX4 at the protein level.

We next revealed the mechanism of CL regulating GPX4 expression. Protein-protein interaction analysis (Fig. S8A) and JASPAR database predicated that C/EBPβ and PPARα may be transcription factors of *Gpx4*. To validate these predictions, we first assessed the role of PPARα. Adipocyte-specific *Ppara* deletion had no effect on *Gpx4* mRNA or GPX4 protein levels (Fig. S8B–D), suggesting PPARα is not a critical regulator of GPX4. In contrast, *Cebpb* knockdown in arterial PVAT-derived adipocytes from normal C57BL/6 mice significantly reduced *Gpx4* mRNA and GPX4 protein levels (Fig. 5B–D). To investigate whether C/EBPβ directly binds to the *GPX4* promoter, we performed CUT&Tag analysis. Compared with negative controls, C/EBPβ showed marked enrichment in the promoter region (−2000 to −1000) (Fig. 5E and F). To further define the binding motif, we generated a series of *GPX4* promoter luciferase reporter constructs containing mutations in the predicted C/EBPβ binding sites, based on the CUT&Tag data and the JASPAR database (Fig. 5G, Fig. S8E–F). C/EBPβ increased luciferase activity relative to the empty vector, whereas mutation of the main binding motif (−1959∼-1949) significantly reduced this activity (Fig. 5H). Collectively, these data indicate that C/EBPβ directly regulates *GPX4* transcription and identify C/EBPβ as a previously unrecognized transcriptional regulator of *GPX4*.

To determine whether CL upregulates C/EBPβ to enhance GPX4 expression, we analyzed C/EBPβ protein levels in arterial PVAT. The protein level of C/EBPβ in arterial PVAT from CL-treated mice was higher than that from saline-treated mice (Fig. 5I and J). Furthermore, we found that CL increased nuclear C/EBPβ abundance, which likely enhances its transcriptional activity (Fig. S9). To verify whether CL increases the transcription of C/EBPβ, the mRNA level of *Cebpb* was measured, and an increase in *Cebpb* mRNA levels occurred in response to CL treatment (Fig. 5K), indicating transcriptional upregulation of C/EBPβ. To further explore whether the regulatory effect of CL on GPX4 is C/EBPβ dependent, *Cebpb* was knocked down prior to CL treatment (1 μM). As expected, CL treatment did not increase GPX4 expression in the absence of *Cebpb*, indicating that the regulatory effect of CL on GPX4 expression is C/EBPβ dependent (Fig. 5L).

β3-AR activation leads to intracellular cyclic adenosine monophosphate (cAMP) accumulation in adipocytes, which in turn activates PKA and CREB signaling pathways to upregulate C/EBPβ expression [[46], [47], [48]]. To investigate whether CL upregulates C/EBPβ through the cAMP-PKA-CREB signaling pathway, we first measured cAMP levels and observed a significant increase following CL (1 μM) treatment (Fig. 5M). We next examined the activation status of PKA and CREB after CL stimulation and found elevated phosphorylation of PKA at Thr197 and CREB at Ser133 (Fig. 5N–P). To further confirm that the regulatory effects of CL are PKA-dependent, we employed Rp-cAMPS, a selective cAMP antagonist that functions as a competitive inhibitor of PKA. As shown in (Fig. 5Q–S), pretreatment with the inhibitor (20 μM) significantly attenuated the CL-induced phosphorylation of both PKA and its downstream target CREB, demonstrating that CL-mediated activation of this signaling axis is indeed dependent on PKA activity. Furthermore, co-treatment with the PKA inhibitor markedly blocked CL-induced upregulation of both *Cebpb* and *Gpx4* (Fig. 5T and U). Collectively, these results demonstrate that CL enhances GPX4 expression by activating the cAMP-PKA-CREB pathway to increase C/EBPβ transcription, establishing C/EBPβ as a critical mediator of CL's anti-ferroptotic effects.

### 3.6 The suppressing role of CL in Ox-LDL-induced ferroptosis in arterial PVAT-derived adipocytes is C/EBPβ dependent

**Fig. 6:**
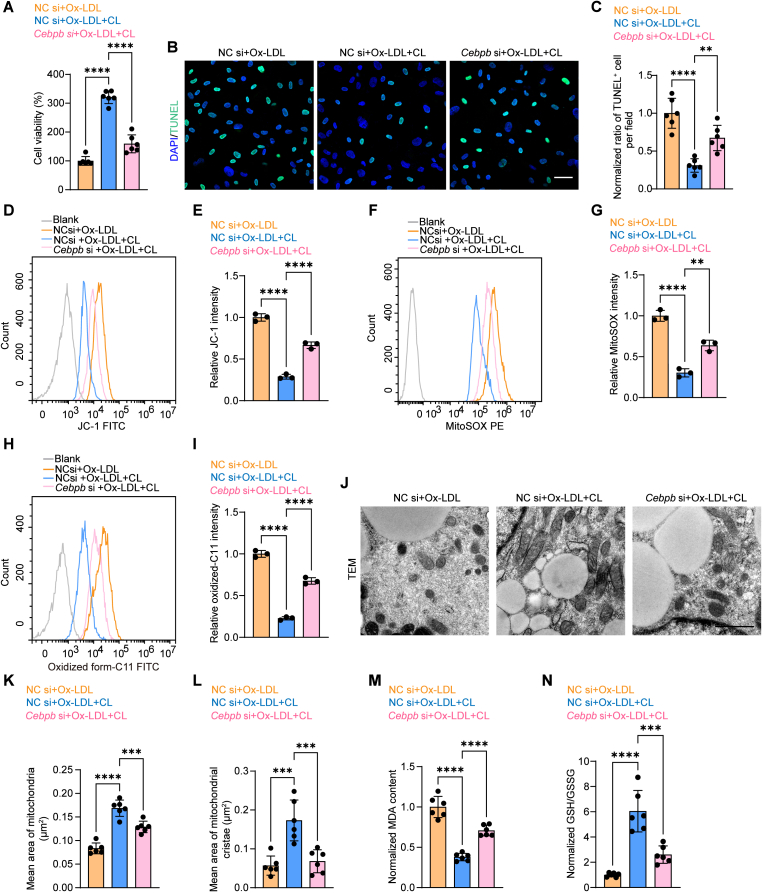
*Cebpb* deficiency abrogates the inhibitory effect of CL-316243 on Ox-LDL-induced adipose ferroptosis in arterial PVAT-derived a**dipocytes.** (A) Normalized values of adipocyte viability as determined by CCK-8 assays (n = 6). Cells were transfected with NC si or *Cebpb* si followed by treatment with Ox-LDL (150 μg/mL) in the presence or absence of CL (1 μM). Forty-eight hours post-siRNA transfection, cells were first pretreated with PBS (as vehicle control) or Ox-LDL for 24 h, followed by a second 24-h treatment with Ox-LDL in combination with either vehicle (DMSO) or CL. n = 3. (B–C) TUNEL assay of arterial PVAT-derived adipocytes under the treatment conditions described in (A). Representative images are shown in (B), and the normalized quantification is shown in (C) (n = 6). Scale bar, 50 μm. (D-E) Analysis of mitochondrial membrane potential in adipocytes under the treatment conditions described in (A), as assessed by flow cytometry. Representative histograms of JC-1 and FITC are shown in (D), and the normalized JC-1 and FITC median fluorescence intensity (MFI) is shown in (E) (n = 3). (F-G) Analysis of mitochondrial ROS in adipocytes under the treatment conditions described in (A), as assessed by flow cytometry. Representative histograms of MitoSOX and PE are shown in (F), and the normalized MitoSOX and PE MFI is shown in (G) (n = 3). (H–I) Oxidized lipid ROS analysis of adipocytes under the treatment conditions described in (A), as assessed by flow cytometry. Representative histograms of the oxidized form of C11 are shown in (H), and the normalized quantification of the MFI is shown in (I) (n = 3). (J-L) Mitochondrial morphology analysis of adipocytes under the treatment conditions described in (A). Representative transmission electron microscopy images of mitochondrial morphology are shown in (J), with quantification of mean area of mitochondria and mean area of mitochondria cristae in (K-L), respectively. Scale bar, 1 μm. (n = 6). (M − N) Determination of normalized MDA content (M) and ratio of GSH/GSSG (N) in adipocytes under the treatment conditions described in (A) (n = 6). Quantification data are shown as the mean ± SD. Differences were assessed using one-way ANOVA with post hoc Tukey's multiple comparisons test. **P < 0.01; ***P < 0.001; ****P < 0.0001. NC, negative control; CL, CL-316243.

Furthermore, to confirm that the suppressing role of CL in Ox-LDL-induced ferroptosis in arterial PVAT-derived adipocytes is C/EBPβ dependent, *Cebpb* siRNA was administered before CL (1 μM) treatment. Cell viability, as measured by CCK-8 assays, was decreased under *Cebpb* knockdown conditions (Fig. 6A). The inhibitory effect of CL on Ox-LDL-induced cell death was also weakened, as indicated by the TUNEL assay (Fig. 6B and C). CL-mediated preservation of mitochondrial membrane potential was reduced in *Cebpb*-deficient cells (Fig. 6D and E). Lipid peroxidation, a hallmark of ferroptosis, was also exacerbated in the absence of *Cebpb*. This was evidenced by increased fluorescence of MitoSOX and oxidized-C11 (Fig. 6F–I). Mitochondrial morphology was also examined, and CL inhibited Ox-LDL-induced mitochondrial shrinkage, whereas *Cebpb* deficiency attenuated this protection (Fig. 6J–L), accompanied with elevated MDA content (Fig. 6M) and reduced GSH/GSSG ratios (Fig. 6N). These findings collectively confirm that CL's ability to suppress Ox-LDL-induced features of ferroptosis in arterial PVAT-derived adipocytes is critically dependent on C/EBPβ.

## 4 Discussion

**Fig. 7:**
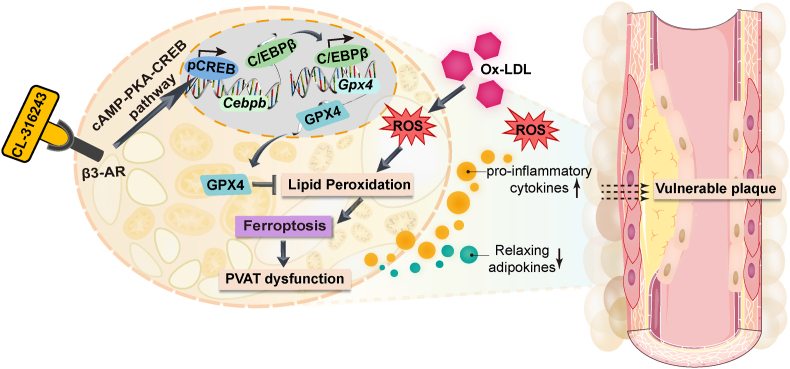
Model for the mechanism of CL-316243 in facilitating stable plaque phenotypes in established athero**sclerosis.** Upon Ox-LDL stimulation, lipid peroxide overload promotes arterial PVAT ferroptosis, thereby leading to arterial PVAT dysfunction, resulting in releasement of vasoactive factors to aggravate vascular inflammation. CL-316243 binds to β3-adrenoceptor and activates cAMP-PKA-CREB signaling pathway to increase C/EBPβ expression, thus transcriptionally promoting the expression of phospholipid hydroperoxidase GPX4, resulting in suppression of arterial PVAT ferroptosis and arterial PVAT dysfunction, consequently reducing vascular inflammation, thereby facilitating stable plaque phenotypes in established atherosclerosis.

Plaque rupture is the primary cause of acute cardiovascular events, yet effective preventive and therapeutic strategies remain limited. PVAT dysfunction contributes to the release of pro-inflammatory cytokines and vasoactive factors, representing a key “outside-to-inside” driver of plaque vulnerability. In the present study, we demonstrate that PVAT ferroptosis is a critical contributor to PVAT dysfunction in established atherosclerosis, and that pharmacological activation of β3-adrenoceptor with CL-316243 enhances stable plaque phenotypes by inhibiting ferroptosis-induced PVAT dysfunction. Mechanistically, CL-316243 upregulates C/EBPβ via the cAMP-PKA-CREB signaling cascade, and we identify C/EBPβ as a novel transcriptional regulator of *GPX4* that directly binds its promoter to drive GPX4 expression and suppress ferroptosis (Fig. 7). Notably, previous studies of CL-316243 in atherosclerosis have largely emphasized systemic lipid lowering and hepatic remnant clearance and have reported no reduction in plaque area, consistent with our observations [44,45,[49], [50], [51], [52]]. However, plaque stability, PVAT function, and ferroptosis were not assessed in those studies. Therefore, our findings establish a PVAT-associated mechanism in which inhibition of PVAT ferroptosis facilitates stable plaque phenotypes, thereby extending prior work on β3-AR agonists and revealing a previously unrecognized regulatory axis. These observations suggest that CL may represent a potential ferroptosis inhibitor and a candidate therapeutic agent for atherosclerotic disease.

High energy intake during atherosclerosis progression increases local oxidative stress and inflammation in adipose tissue, resulting in arterial PVAT dysfunction. Dysfunctional arterial PVAT promotes infiltration of inflammatory immune cells, local oxidative stress, and release of PVAT-derived vasoactive factors, thereby triggering an “outside-to-inside” pathological signaling to aggravate atherosclerosis. However, the regulatory mechanism of PVAT dysfunction in established atherosclerosis remains unclear. In the present study, we identified ferroptosis in PVAT at established atherosclerotic plaque, indicated by increased mitochondrial dysfunction, oxidative stress and excessive lipid peroxidation in PVAT, contributing to PVAT dysfunction (Fig. 1). Although ferroptosis has been identified in endothelial cells, smooth muscle cells, and macrophages, and has been demonstrated to promote atherosclerosis, its identification in PVAT is reported here for the first time, indicating a novel pathogenic mechanism for atherosclerosis. Moreover, ferroptosis in multiple vascular cell types during atherosclerosis suggests that ferroptosis serves as a critical pro-atherogenic pathological factor. In the clinic, pericoronary adipose tissue was shown to store and supply Ox-LDL in human coronary plaques [40], indicating the large amount of Ox-LDL in arterial PVAT may be an important factor for ferroptosis during atherosclerosis, which was confirmed by our *in vitro* assays. Our results demonstrated that PVAT ferroptosis could be induced by Ox-LDL treatment, which is further confirmed by ferroptosis inhibitor Fer-1 (Fig. 2A–I). Taken together, our finding suggest that this Ox-LDL-ferroptosis-PVAT dysfunction axis provides a novel pathogenic mechanism for atherosclerosis. Surprisingly, in addition to ferroptosis, other types of cell death have not yet been revealed in arterial PVAT during atherogenesis. Therefore, it will be interesting to explore whether there are other forms of cell death in PVAT during atherogenesis.

In our study, we found that CL treatment inhibits PVAT ferroptosis by reducing mitochondrial damage, mitochondrial ROS, excessive lipid peroxidation, and adipocyte redox imbalance (Fig. 2). Consequently, CL restored PVAT function, including increased expression of UCP1 and PGC1-α, reduced releasement of pro-inflammatory cytokines, as well as reduced expression of pro-inflammatory genes than the control groups (Fig. 3). These effects contributed to stable plaque phenotypes in established atherosclerosis as indicated by reduced necrotic core area, CD68 filtration, MMP9 level, and increased collagen content (Fig. 4). Notably, these plaque stability indicators correlated significantly with MDA content and the GSH/GSSG ratio in corresponding PVAT (Fig. S5). These findings not only confirm the relationship between PVAT dysfunction and stable plaque phenotypes, but also suggest that maintaining PVAT homeostasis may represent a potential strategy for facilitating stable plaque phenotypes. The association between PVAT function and atherosclerotic plaque stability may be mediated by PVAT-derived factors that act on vascular smooth muscle cells (VSMCs) or myofibroblasts. Under physiological conditions, PVAT functions as a paracrine organ that transduces metabolic signals to blood vessels to maintain cellular homeostasis [53]. Notably, visfatin and adiponectin are identified as PVAT-derived adipokines for proliferation and migration of VSMCs [54,55]. VSMCs and their phenotypic derivatives, myofibroblasts, serve as the builders and maintainers of stable atherosclerotic plaques in the vessel wall and are capable of extensively producing extracellular matrix components such as collagen, elastic fibers, and proteoglycans, which form a dense, sturdy fibrous cap [56]. Moreover, by secreting anti-inflammatory factors such as TGF-β and tissue inhibitors of metalloproteinases, they help suppress excessive plaque inflammation and counteract the fibrous cap-degrading activity of pro-inflammatory macrophage-derived MMPs [57,58]. Under pathological conditions, dysfunctional PVAT shifts toward a pro-inflammatory phenotype, releasing adipokines (MCP-1, TNF-α, IL-6 and PAI-1) and ROS that promote plaque instability via “outside-inside” way [59]. Sustained inflammation and oxidative stress impair the activity of smooth muscle cells and myofibroblasts, leading to cellular senescence and apoptosis. Consequently, collagen synthesis declines while pro-inflammatory signaling increases, leading to fibrous cap thinning, necrotic core expansion, and ultimately the progression to a high-risk vulnerable plaque [60,61]. Therefore, CL could be a new approach to prevent plaque rupture by improving PVAT function. Surprisingly, CL has no effect on atherosclerotic plaque area, indicating improvement of PVAT function may be not enough to inhibit atherosclerosis in this *Apoe*^−/−^ mouse model. This may be because CL does not have a significant lipid-lowing effect in this model, which is consistent with other's study [45].

Ferroptosis is regulated by lipid peroxidation and iron metabolism related genes, such as *GPX4*, *NRF2*, *SLC7A11*, *ATF4*, *FSP1*, and *TFR1* [39]. To further determine the mechanism by which CL inhibits ferroptosis, we analyzed the expression of these ferroptosis associated genes after CL treatment and found that CL could significantly upregulate the mRNA and protein level of GPX4 (Fig. 5I–K). In the vascular tissue of atherosclerotic mice, the expression level of lipid peroxide scavenger GPX4 was significantly reduced [62], suggesting its critical role in atherosclerosis. In previous studies, several transcription factors of *GPX4* in certain cell types have been identified, such as PPARα in NCTC 1469 cells and neuronal cells, ELK in endothelial cells, and the AP-1 protein Jun as a transcriptional suppressor in pancreatic acinar cells [[63], [64], [65]]. However, the major transcription factor that regulates *GPX4* in adipocytes is still unknown. Through bioinformatics analysis and experimental verification, we showed for the first time that C/EBPβ is a novel transcription factor of *GPX4* in adipocytes. Furthermore, we proved that C/EBPβ could directly bind to the promotor of *GPX4* (Fig. 5E–H). Interestingly, PPARα was also identified as a candidate by our bioinformatics analysis, but knockdown of PPARα did not affect the mRNA level of *Gpx4*, suggesting that PPARα does not play a dominant role in *Gpx4* transcription in adipocytes. Further investigation showed that CL treatment upregulated C/EBPβ expression, but failed to reverse Ox-LDL-induced mitochondrial dysfunction, oxidative stress and excessive lipid peroxidation in *Cebpb*-deficient arterial PVAT-derived adipocytes (Fig. 5, Fig. 6), suggesting the suppressing role of CL in Ox-LDL-induced ferroptosis in arterial PVAT-derived adipocytes is C/EBPβ dependent. Interestingly, the role of C/EBPβ in different cell types are complex. For instance, in macrophages and endothelial cells, C/EBPβ mediates inflammatory cytokine expression in conjunction with NF-κB; moreover, it is a major driver of IL-6 expression [66], which promote local inflammation. Thus, specifically increasing C/EBPβ in adipocytes may be a good option for cardiovascular disease treatment. In this regard, CL has a natural advantage as its binding receptor is mainly expressed in adipocytes. Therefore, CL may represent a potential ferroptosis inhibitor with specificity for adipose tissue and could offer cardiovascular benefits in preclinical models.

There remain some limitations in our study. First, our design has limitations in the clinical conclusions that can be drawn, and the mechanism of CL suggested here should be further examined in clinical samples. Additionally, our data showed that CL enhanced arterial PVAT function by increasing GPX4; however, more solid conclusions need to be obtained by using GPX4 inhibitor. Furthermore, the precise downstream signaling events that link ferroptosis suppression in PVAT to structural plaque stabilization, such as potential effects on efferocytosis, TGF-β-mediated fibrosis, or NF-κB activity, remain to be investigated. In the present study, we focused on stable plaque phenotypes as the primary outcome. Whether CL-induced suppression of PVAT ferroptosis also subsequently modulates vascular relaxation or local redox status remains to be investigated. Future studies using aortic ring assays and vascular Dihydroethidium staining will help address this question. Finally, due to technical constraints, we were unable to perform truly localized perivascular delivery. Although our *ex vivo* PVAT experiments suggest that PVAT dysfunction may contribute to plaque instability, the systemic drug administration and the complexity of the *in vivo* model make it difficult to determine whether local PVAT effects are the primary cause. Given that β3-adrenoceptors are far more abundant in brown adipose tissue, such as PVAT, than in white adipose tissue [67,68], PVAT is likely a major target of CL; however, contributions from systemic BAT activation or WAT browning or systemic metabolic also changes cannot be entirely ruled out. Future studies employing PVAT-targeted drug delivery will be essential to conclusively establish the causal role of CL to PVAT-intrinsic ferroptosis in atherosclerosis.

In conclusion, this study identifies for the first time the involvement of PVAT ferroptosis at established atherosclerotic plaque and reveals CL as a specific agent to increase stable plaque phenotypes by suppressing arterial PVAT ferroptosis in a C/EBPβ-dependent way. On the molecular mechanism, we found that CL transcriptionally promotes the expression of ferroptosis suppressor GPX4 via activation of the β3-AR-cAMP-PKA-CREB-C/EBPβ signaling pathway. These findings highlight the importance of adipose tissue redox balance in cardiovascular disease and suggest that improving PVAT function may be an effective strategy to facilitate stable plaque phenotypes in atherosclerosis. By demonstrating that CL-316243 mitigates PVAT ferroptosis through the C/EBPβ-GPX4 axis, our study identifies this β3-adrenergic agonist as a promising pharmacological intervention to stabilize atherosclerotic plaques and reduce cardiovascular risk.

## CRediT authorship contribution statement

**Yuanqing Jiang:** Investigation, Writing – original draft. **Yi Li:** Investigation, Writing – original draft. **Kefan Ma:** Investigation. **Suxiang Guo:** Investigation. **Nachuan Liao:** Investigation. **Jiayi Zhou:** Investigation. **Junbo Chen:** Investigation. **Ruizhe Ren:** Writing – review & editing. **Yaohui Kou:** Writing – review & editing. **Jinying Li:** Writing – review & editing. **He Liu:** Writing – review & editing. **Yang Wei:** Investigation. **Xiaofei Zhou:** Writing – review & editing. **Linge Fan:** Writing – review & editing. **Lingfeng Qin:** Writing – review & editing. **Haige Zhao:** Funding acquisition. **Ying Xiao:** Writing – review & editing. **Luyang Yu:** Funding acquisition, Writing – review & editing. **Zhen Ge:** Funding acquisition, Supervision, Writing – review & editing. **Cong Qiu:** Funding acquisition, Project administration, Supervision, Writing – review & editing.

## Declaration of competing interest

The authors declare no competing interest.

## Data Availability

Data will be made available on request.

## Appendix A Supplementary data

Fig. S1. Gene Set Enrichment Analysis (GSEA) analysis of differentially expressed genes (DEGs) in in PVAT from control mice and atheroscler**otic mice.** GSEA plot and gene sets for inflammatory response (A), chemokine mediated signaling pathway (B), fatty acid metabolic process (C), and cAMP mediated signaling pathway (D) in PVAT from control mice (Ctrl) and atherosclerotic mice (AS), according to the results of RNA-seq. NES, ES, P-value and FDR value (FDR-corrected P-value) are presented. Fig. S2. GO and KEGG enrichment analysis of differentially expressed genes (DEGs) in high-fat diet (HFD)-treated PVAT and standard fat diet (SFD)-treated PVAT. (A) The GO enrichment bubble plot showing the DEGs of HFD arterial PVAT versus SFD arterial PVAT was determined by GEO dataset analysis (C57BL/6J mice, n = 3 per group). (B) KEGG enrichment bubble plot showing the DEGs of HFD arterial PVAT vs. SFD arterial PVAT was determined by GEO dataset analysis (C57BL/6J mice, n = 3 per group). All raw data were collected from Gene expression omnibus (GEO) database contained SFD-fed or HFD-fed C57BL/6J mice arterial PVAT RNA sequencing samples (GSE28440). Fig. S3. Correlation analyses between ferroptosis-related markers and PVAT dysfunction-related markers in PVAT. (A) Correlation between MDA levels and relative mRNA levels of Il1b. (B) Correlation between MDA levels and relative mRNA levels of Lep. (C) Correlation between GSH/GSSG and relative mRNA levels of Il1b. (D) Correlation between GSH/GSSG and relative mRNA levels of Lep. (E) Correlation between relative mRNA levels of Acsl4 and Il1b. (F) Correlation between relative mRNA levels of Acsl4 and Lep. (G) Correlation between relative mRNA levels of Slc7a11 and Il1b. (H) Correlation between relative mRNA levels of Slc7a11 and Lep. (I) Correlation between relative mRNA levels of Gpx4 and Il1b. (J) Correlation between relative mRNA levels of Gpx4 and Lep. Correlation analyses were conducted using two-tailed Pearson correlation analysis. Fig. S4. ROS scavenger rescues Ox-LDL induced ferroptosis and dysfunction *in vitro*. (A-C) Measurement of normalized Fe^2+^ content (A), MDA (B), GSH/GSSG ratio (C) in arterial PVAT-derived adipocytes treated by PBS, Ox-LDL (150 μg/mL) or Ox-LDL (150 μg/mL) plus ROS scavenger NAC (5 μM). Cells were first pretreated with PBS (as vehicle control) or Ox-LDL for 24 h, followed by a second 24-h treatment with Ox-LDL in combination with either vehicle (DMSO) or NAC. n = 6. (D-I) mRNA levels of genes associated with ferroptosis and PVAT dysfunction in PVAT-derived adipocytes treated as described in (A). n = 6. (J-K) ELISA analysis of TNF-α, IL-1β and IL-6 levels in the conditioned medium of PVAT-derived adipocytes treated as described in (A). n = 6. The quantification data are from at least 6 independent biological samples per group and are shown as the mean ± SD. Differences were analyzed by one-way ANOVA with post hoc Tukey's multiple comparisons test. *P < 0.05, **P < 0.01, ***P < 0.001, ****P < 0.0001. Ctrl, Control; Ox-LDL, Oxidized low-density lipoprotein; NAC, N-Acetylcysteine. Fig. S5. Correlation of ferroptosis markers (MDA and GSH/GSSG ratio) with atherosclerotic plaque vulnerability markers. (A-D) Correlation analysis between MDA content and necrotic core area (A), collagen content (B), CD68-positive area (C), and MMP-positive area (D). (E-H) Correlation analysis between GSH/GSSG and necrotic core area (E), collagen content (F), CD68-positive area (G), and MMP-positive area (H). Correlation analyses were conducted using two-tailed Pearson correlation analysis. Fig. S6. Effects of CL-316243 on plasma lipid profiles, blood pressure, and heart rate in mice. (A) total cholesterol (TC), (B) low-density lipoprotein-cholesterol (LDL-C), and (C) and high-density lipoprotein-cholesterol (HDL-C) were detected from plasma in saline or CL (1 mg/kg) treated Apoe−/− mice (n = 6). (D-F) Systolic blood pressure (D), diastolic blood pressure (E), and heart rate (F) in Apoe^−/−^ mice treated with saline or CL (1 mg/kg) for 4 weeks (n = 6). Data are presented as mean ± SD. Differences were analyzed by unpaired *t*-test. *P < 0.05; ns, not significant. CL, CL-316243. Fig. S7. CL treatment upregulates GPX4 protein levels. (A-B) Western blotting analysis of GPX4 protein expression in arterial PVAT from saline- or CL-treated mice. Representative blots are shown in (A) with normalized quantification in (B). (C–F) Histological analysis of GPX4 in tPVAT and aPVAT from saline- or CL-treated Apoe^−/−^ mice. Representative IHC images of GPX4 are shown in (C, E) with the quantification of the average optical density (AOD) of GPX4 in (D, F). Scale bar, 50 μm. (n = 6). Quantification data are shown as the mean ± SD. Differences were assessed using unpaired *t*-test. ****P < 0.0001. CL, CL-316243; tPVAT, thoracic aortic PVAT; aPVAT, abdominal aortic PVAT. Fig. S8. Predicted transcriptional motifs in the GPX4 promoter. (A) Protein-protein interaction network analysis of the ferroptosis suppressor GPX4 with C/EBPβ and PPARα. (B) Relative mRNA level of Gpx4 in arterial PVAT-derived adipocytes transfected with negative control (NC si) or Ppara si (n = 6). (C-D) WB analysis of GPX4 protein expression in adipocytes after transfection with NC si or Ppara si. Representative blots are shown in (C), and the normalized quantification is shown in (D) (n = 6). (E) Predicted scores and locations of potential C/EBPβ-regulated motifs within the GPX4 promoter via the JASPAR Database. (F) Schematic diagram of GPX4 promoter plasmid construction containing motif mutations. Values are shown as the mean ± SD. Differences were analyzed by unpaired *t*-test. NC, negative control; ns, not significant. Fig. S9. CL-316243 increases C/EBPβ expression in the nucleus. (A) Representative blots of C/EBPβ in nuclear and cytoplasmic fractions and whole lysate of arterial PVAT adipocytes after vehicle (DMSO) or CL treatment (1 μM). (B) Normalized ration of nuclear/cytoplasmic C/EBPβ. The data are shown as the mean ± SD, n = 6, unpaired *t-*test. ****P < *0.001. CL, CL-316243.Multimedia Component 1

## References

[1] Libby P. (2021). The changing landscape of atherosclerosis. Nature.

[2] Prati F., Romagnoli E., Gatto L. (2020). Relationship between coronary plaque morphology of the left anterior descending artery and 12 months clinical outcome: the clima study. Eur. Heart J..

[3] Virmani R., Burke A.P., Farb A. (2006). Pathology of the vulnerable plaque. J. Am. Coll. Cardiol..

[4] Zhang Y.Y., Shi Y.N., Zhu N. (2021). Pvat targets vsmcs to regulate vascular remodelling: Angel or demon. J. Drug Target..

[5] Grigoras A., Amalinei C., Balan R.A. (2019). Perivascular adipose tissue in cardiovascular diseases-an update. Anatol. J. Cardiol..

[6] Sowka A., Dobrzyn P. (2021). Role of perivascular adipose tissue-derived adiponectin in vascular homeostasis. Cells.

[7] Katsiki N., Mikhailidis D.P. (2022). Perivascular adipose tissue: pathophysiological links with inflammation, atherosclerosis, and thrombosis. Angiology.

[8] Qi X.Y., Qu S.L., Xiong W.H. (2018). Perivascular adipose tissue (pvat) in atherosclerosis: a double-edged sword. Cardiovasc. Diabetol..

[9] Hu H., Garcia-Barrio M., Jiang Z.S. (2021). Roles of perivascular adipose tissue in hypertension and atherosclerosis. Antioxidants Redox Signal..

[10] Stanek A., Brozyna-Tkaczyk K., Myslinski W. (2021). The role of obesity-induced perivascular adipose tissue (pvat) dysfunction in vascular homeostasis. Nutrients.

[11] Horimatsu T., Patel A.S., Prasad R. (2018). Remote effects of transplanted perivascular adipose tissue on endothelial function and atherosclerosis. Cardiovasc. Drugs Ther..

[12] Kim S., Lee E.S., Lee S.W. (2019). Site-specific impairment of perivascular adipose tissue on advanced atherosclerotic plaques using multimodal nonlinear optical imaging. Proc. Natl. Acad. Sci. U. S. A..

[13] Tan N., Dey D., Marwick T.H. (2023). Pericoronary adipose tissue as a marker of cardiovascular risk: jacc review topic of the week. J. Am. Coll. Cardiol..

[14] Hertzel A.V., Yong J., Chen X. (2022). Immune modulation of adipocyte mitochondrial metabolism. Endocrinology.

[15] Deng L., He S., Guo N. (2023). Molecular mechanisms of ferroptosis and relevance to inflammation. Inflamm. Res..

[16] Bai T., Li M., Liu Y. (2020). Inhibition of ferroptosis alleviates atherosclerosis through attenuating lipid peroxidation and endothelial dysfunction in mouse aortic endothelial cell. Free Radic. Biol. Med..

[17] Li L., Wang H., Zhang J. (2021). Effect of endothelial progenitor cell-derived extracellular vesicles on endothelial cell ferroptosis and atherosclerotic vascular endothelial injury. Cell Death Discov..

[18] Yang K., Song H., Yin D. (2021). Pdss2 inhibits the ferroptosis of vascular endothelial cells in atherosclerosis by activating nrf2. J. Cardiovasc. Pharmacol..

[19] Bao X., Luo X., Bai X. (2023). Cigarette tar mediates macrophage ferroptosis in atherosclerosis through the hepcidin/fpn/slc7a11 signaling pathway. Free Radic. Biol. Med..

[20] Liu W., Ostberg N., Yalcinkaya M. (2022). Erythroid lineage jak2v617f expression promotes atherosclerosis through erythrophagocytosis and macrophage ferroptosis. J. Clin. Investig..

[21] You J., Ouyang S., Xie Z. (2023). The suppression of hyperlipid diet-induced ferroptosis of vascular smooth muscle cells protests against atherosclerosis independent of p53/scl7a11/gpx4 axis. J. Cell. Physiol..

[22] Guo Z., Zhang W., Gao H. (2024). High expression levels of haem oxygenase-1 promote ferroptosis in macrophage-derived foam cells and exacerbate plaque instability. Redox Biol..

[23] Rong J., Li C., Zhang Q. (2023). Hydroxysafflor yellow a inhibits endothelial cell ferroptosis in diabetic atherosclerosis mice by regulating mir-429/slc7a11. Pharm. Biol..

[24] Zhao Y., Zhao Y., Tian Y. (2022). Metformin suppresses foam cell formation, inflammation and ferroptosis via the ampk/erk signaling pathway in ox-ldl-induced thp-1 monocytes. Exp. Ther. Med..

[25] Luo X., Wang Y., Zhu X. (2024). Mcl attenuates atherosclerosis by suppressing macrophage ferroptosis via targeting keap1/nrf2 interaction. Redox Biol..

[26] Man A.W.C., Zhou Y., Xia N. (2020). Perivascular adipose tissue as a target for antioxidant therapy for cardiovascular complications. Antioxidants.

[27] Cheng C.K., Ding H., Jiang M. (2023). Perivascular adipose tissue: Fine-tuner of vascular redox status and inflammation. Redox Biol..

[28] Advanced ScienceGao M., Yi J., Zhu J. (2019). Role of mitochondria in ferroptosis. Mol. Cell.

[29] Wang B., Wang Y., Zhang J. (2023). Ros-induced lipid peroxidation modulates cell death outcome: mechanisms behind apoptosis, autophagy, and ferroptosis. Arch. Toxicol..

[30] Weyer C., Tataranni P.A., Snitker S. (1998). Increase in insulin action and fat oxidation after treatment with cl 316,243, a highly selective beta3-adrenoceptor agonist in humans. Diabetes.

[31] Arch J.R. (2002). Beta(3)-adrenoceptor agonists: potential, pitfalls and progress. Eur. J. Pharmacol..

[32] Shin W., Okamatsu-Ogura Y., Matsuoka S. (2019). Impaired adrenergic agonist-dependent beige adipocyte induction in obese mice. J. Vet. Med. Sci..

[33] Valentine J.M., Ahmadian M., Keinan O. (2022). Beta3-adrenergic receptor downregulation leads to adipocyte catecholamine resistance in obesity. J. Clin. Investig..

[34] Ying R., Li S.W., Chen J.Y. (2018). Endoplasmic reticulum stress in perivascular adipose tissue promotes destabilization of atherosclerotic plaque by regulating gm-csf paracrine. J. Transl. Med..

[35] Kip P., Sluiter T.J., MacArthur M.R. (2024). Preoperative methionine restriction induces perivascular adipose tissue browning and improves vein graft remodeling in male mice. Nat. Commun..

[36] Victorio J.A., Barssotti L., Aprahamian T. (2024). Beta-adrenergic stimulation-induced pvat dysfunction in male sex: a role for 11beta-hydroxysteroid dehydrogenase-1. Endocrinology.

[37] Withers S.B., Forman R., Meza-Perez S. (2017). Eosinophils are key regulators of perivascular adipose tissue and vascular functionality. Sci. Rep..

[38] Liang M., Huang Y., Jiang Y. (2023). Isolation, culture, and adipogenic induction of stromal vascular fraction-derived preadipocytes from mouse periaortic adipose tissue. J. Vis. Exp..

[39] Zhang S., Sun Z., Jiang X. (2022). Ferroptosis increases obesity: crosstalk between adipocytes and the neuroimmune system. Front. Immunol..

[40] Uchida Y., Uchida Y., Shimoyama E. (2017). Human pericoronary adipose tissue as storage and possible supply site for oxidized low-density lipoprotein and high-density lipoprotein in coronary artery. J. Cardiol..

[41] You Z., Ye X., Jiang M. (2023). Lnc-mrgprf-6:1 promotes ox-ldl-induced macrophage ferroptosis via suppressing gpx4. Mediat. Inflamm..

[42] Jiang Y., Gong F. (2023). Immune cells in adipose tissue microenvironment under physiological and obese conditions. Endocrine.

[43] Nicholls D.G. (2021). Mitochondrial proton leaks and uncoupling proteins. Biochim. Biophys. Acta Bioenerg..

[44] Worthmann A., Schlein C., Berbee J.F.P. (2019). Effects of pharmacological thermogenic adipocyte activation on metabolism and atherosclerotic plaque regression. Nutrients.

[45] Berbee J.F., Boon M.R., Khedoe P.P. (2015). Brown fat activation reduces hypercholesterolaemia and protects from atherosclerosis development. Nat. Commun..

[46] Liu H., Mei M., Lin S. (2025). Wuling san regulates avpr2-camp-pka-creb pathway to delay cellular senescence and ameliorate acute kidney injury. J. Ethnopharmacol..

[47] Xiao Y., Wang J., Zhang H. (2025). Linarin alleviates high-fat diet-induced hepatic steatosis by inhibiting pde4d and activating the camp/pka/creb pathway. Free Radic. Biol. Med..

[48] Zhang J.W., Klemm D.J., Vinson C. (2004). Role of creb in transcriptional regulation of ccaat/enhancer-binding protein beta gene during adipogenesis. J. Biol. Chem..

[49] Zhou E., Hoeke G., Li Z. (2020). Colesevelam enhances the beneficial effects of brown fat activation on hyperlipidaemia and atherosclerosis development. Cardiovasc. Res..

[50] Zhou E., Li Z., Nakashima H. (2021). Hepatic scavenger receptor class b type 1 knockdown reduces atherosclerosis and enhances the antiatherosclerotic effect of brown fat activation in apoe*3-leiden.Cetp mice. Arterioscler. Thromb. Vasc. Biol..

[51] Zhou E., Li Z., Nakashima H. (2021). Beneficial effects of brown fat activation on top of pcsk9 inhibition with alirocumab on dyslipidemia and atherosclerosis development in apoe*3-leiden.Cetp mice. Pharmacol. Res..

[52] Wang Y., Wang Y., Jiang H.F. (2022). Mirabegron ameliorated atherosclerosis of apoe(-/-) mice in chronic intermittent hypoxia but not in normoxia. Cardiovasc. Drugs Ther..

[53] Grodecki K., Geers J., Kwiecinski J. (2025). Phenotyping atherosclerotic plaque and perivascular adipose tissue: signalling pathways and clinical biomarkers in atherosclerosis. Nat. Rev. Cardiol..

[54] Wang P., Xu T.Y., Guan Y.F. (2009). Perivascular adipose tissue-derived visfatin is a vascular smooth muscle cell growth factor: role of nicotinamide mononucleotide. Cardiovasc. Res..

[55] Takaoka M., Nagata D., Kihara S. (2009). Periadventitial adipose tissue plays a critical role in vascular remodeling. Circ. Res..

[56] Bennett M.R., Sinha S., Owens G.K. (2016). Vascular smooth muscle cells in atherosclerosis. Circ. Res..

[57] Grootaert M.O.J., Bennett M.R. (2021). Vascular smooth muscle cells in atherosclerosis: time for a re-assessment. Cardiovasc. Res..

[58] Jarad S., Gill G., Amadi P. (2025). Vsmcs in atherosclerosis: implications on the role of inflammation and extracellular matrix remodelling. Pharmacol. Res..

[59] Takaoka M., Suzuki H., Shioda S. (2010). Endovascular injury induces rapid phenotypic changes in perivascular adipose tissue. Arterioscler. Thromb. Vasc. Biol..

[60] Chen Y., Qin Z., Wang Y. (2021). Role of inflammation in vascular disease-related perivascular adipose tissue dysfunction. Front. Endocrinol..

[61] Yan A., Gotlieb A.I. (2023). The microenvironment of the atheroma expresses phenotypes of plaque instability. Cardiovasc. Pathol..

[62] Guo Z., Ran Q., Roberts L.J. (2008). Suppression of atherogenesis by overexpression of glutathione peroxidase-4 in apolipoprotein e-deficient mice. Free Radic. Biol. Med..

[63] Qu X.X., He J.H., Cui Z.Q. (2022). Ppar-alpha agonist gw7647 protects against oxidative stress and iron deposit via gpx4 in a transgenic mouse model of alzheimer's diseases. ACS Chem. Neurosci..

[64] Ma X., Dong X., Xu Y. (2022). Identification of ap-1 as a critical regulator of glutathione peroxidase 4 (gpx4) transcriptional suppression and acinar cell ferroptosis in acute pancreatitis. Antioxidants.

[65] Wei S., Yu Z., Shi R. (2022). Gpx4 suppresses ferroptosis to promote malignant progression of endometrial carcinoma via transcriptional activation by elk1. BMC Cancer.

[66] Billack B., Heck D.E., Mariano T.M. (2002). Induction of cyclooxygenase-2 by heat shock protein 60 in macrophages and endothelial cells. Am. J. Physiol.: Cell Physiol..

[67] Virtanen K.A., Lidell M.E., Orava J. (2009). Functional brown adipose tissue in healthy adults. N. Engl. J. Med..

[68] Zhao J., Unelius L., Bengtsson T. (1994). Coexisting beta-adrenoceptor subtypes: significance for thermogenic process in brown fat cells. Am. J. Physiol..

