# Supplementary figures and images for: CL-316243 facilitates stable atherosclerotic plaque phenotypes in association with suppression of perivascular adipose tissue ferroptosis via upregulating C/EBPβ
Source: Redox Biol. 2026 Jun 13;95:104259. doi: 10.1016/j.redox.2026.104259 (PMC13279912; doi:10.1016/j.redox.2026.104259)

**Fig. S1**

**A**

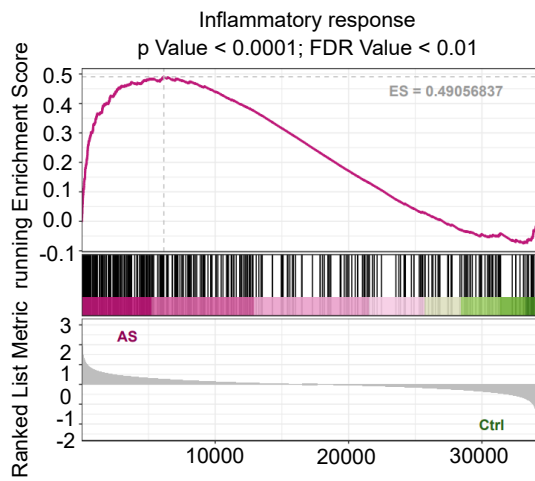

**B**

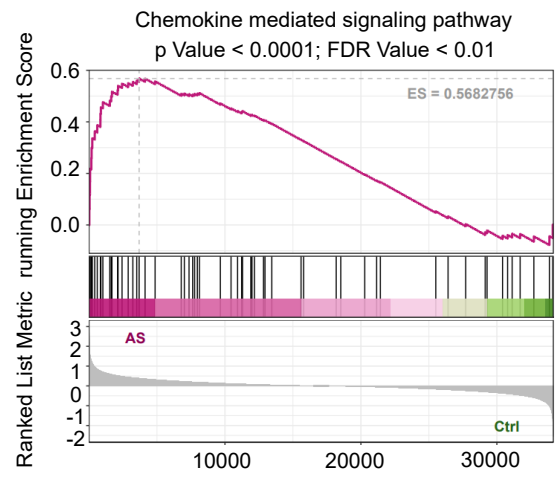

**C**

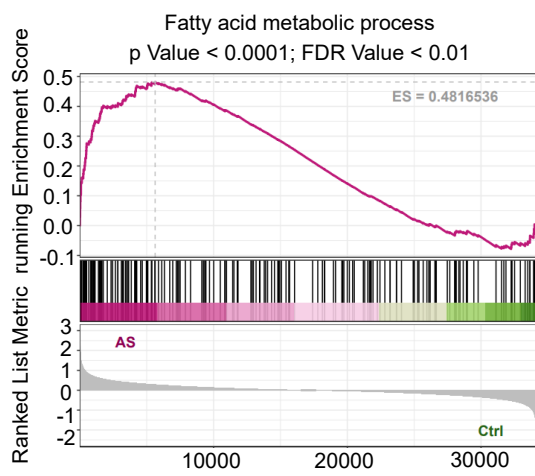

**D**

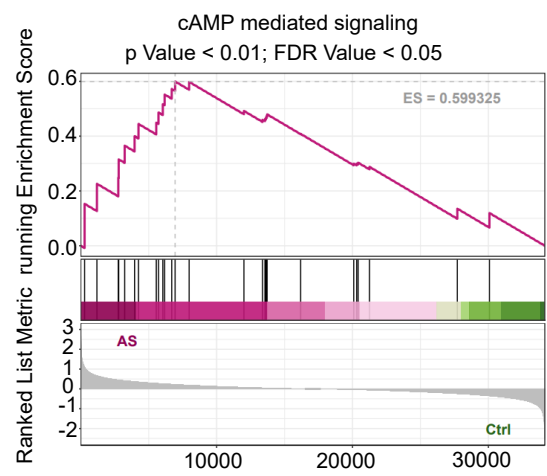

Fig. S2

A

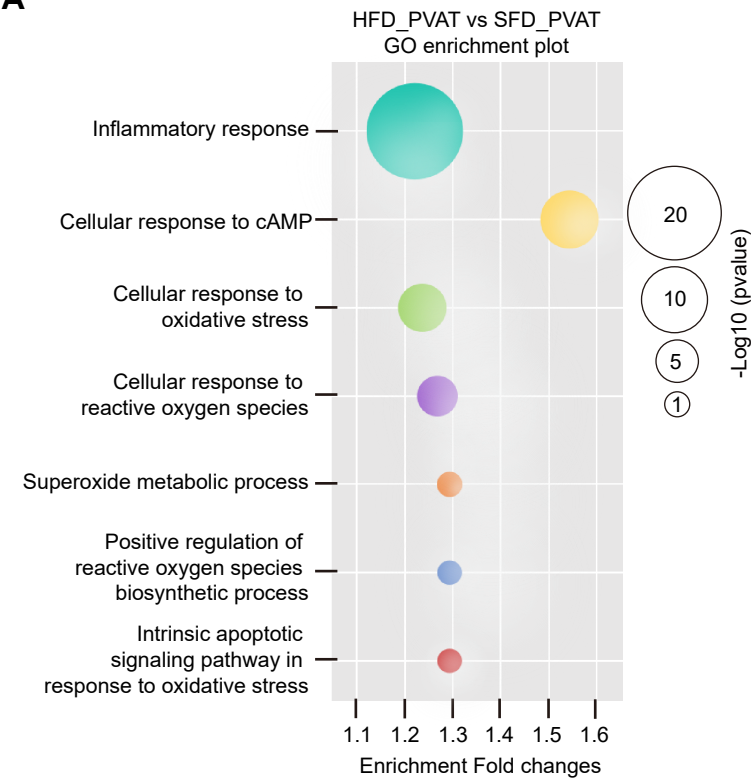

B

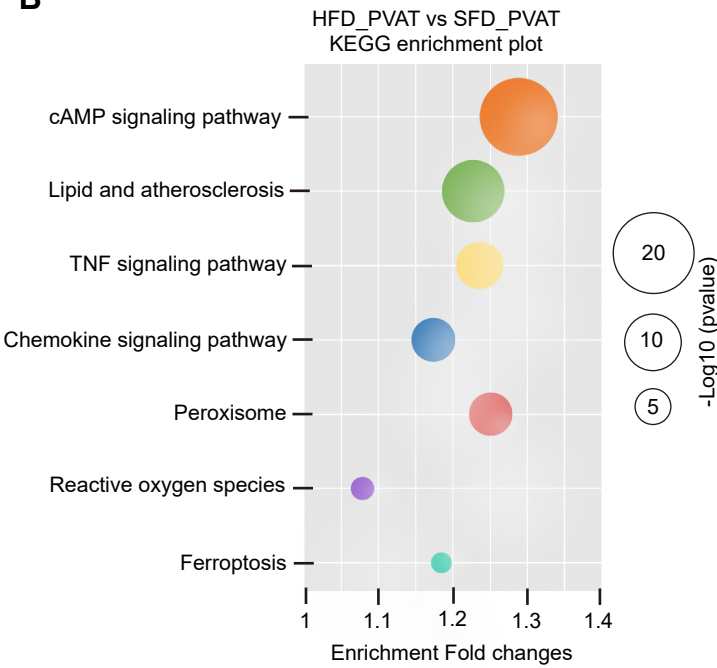

**Fig. S3**

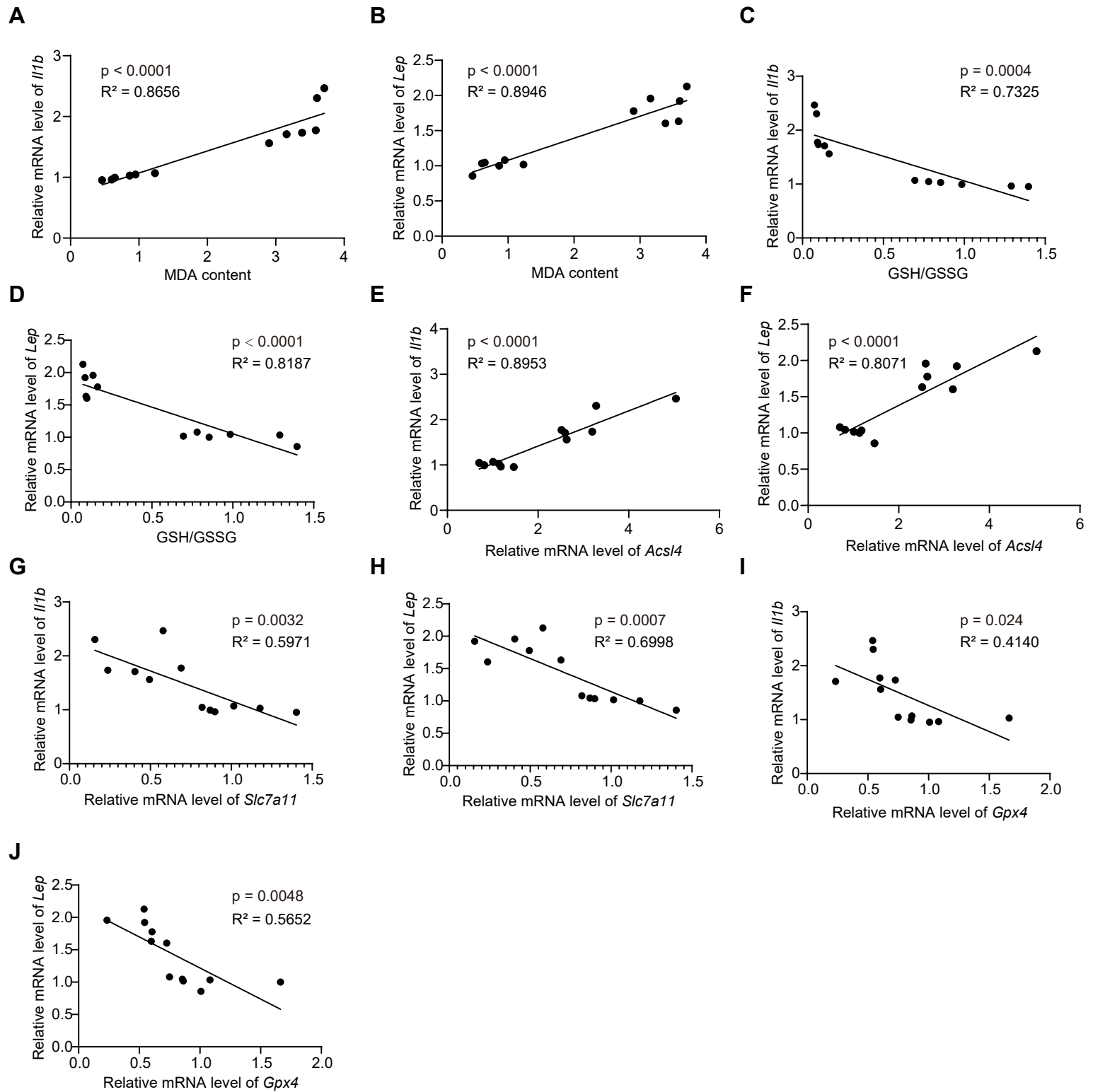

**Fig. S4**

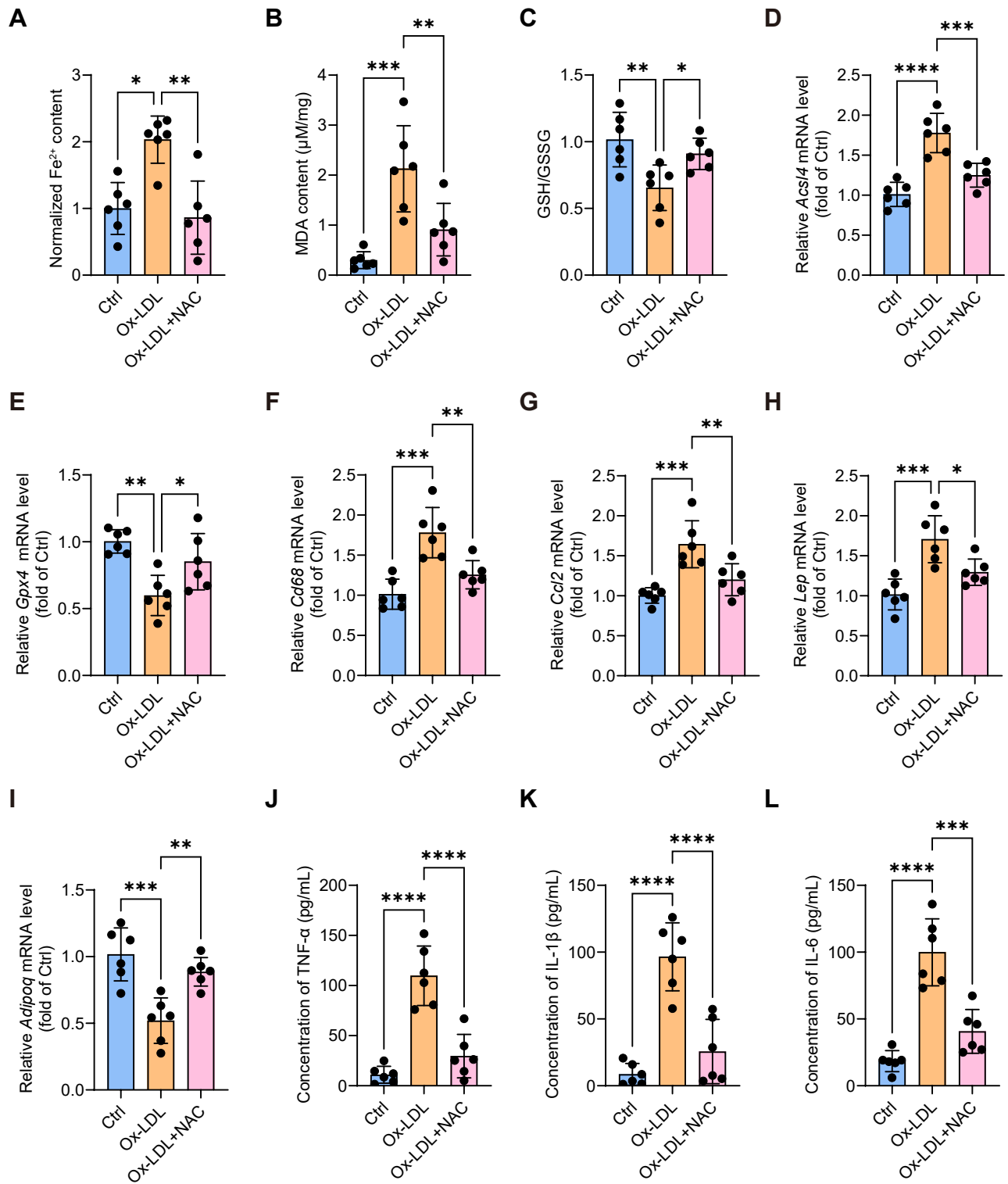

Fig. S5

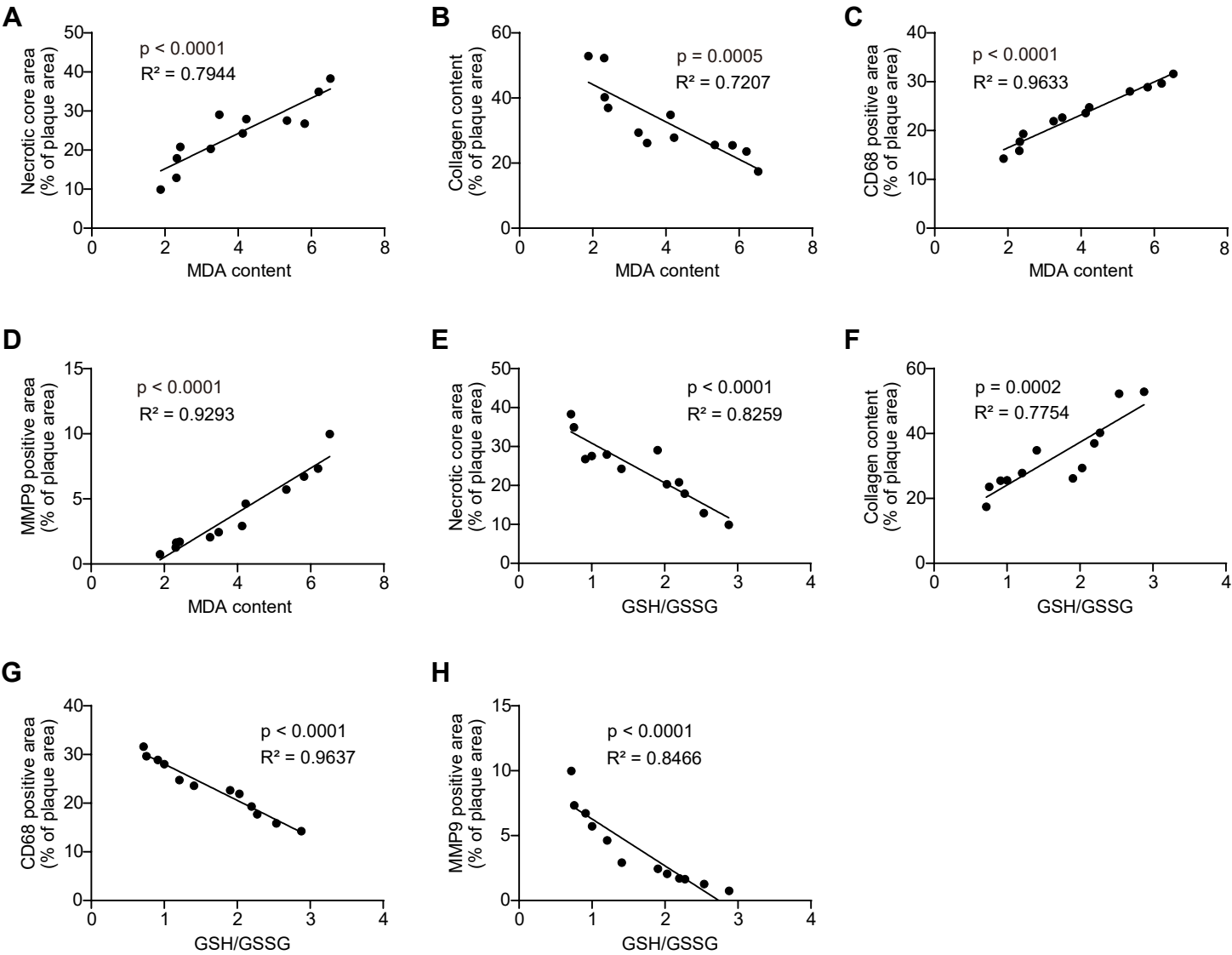

Fig. S6

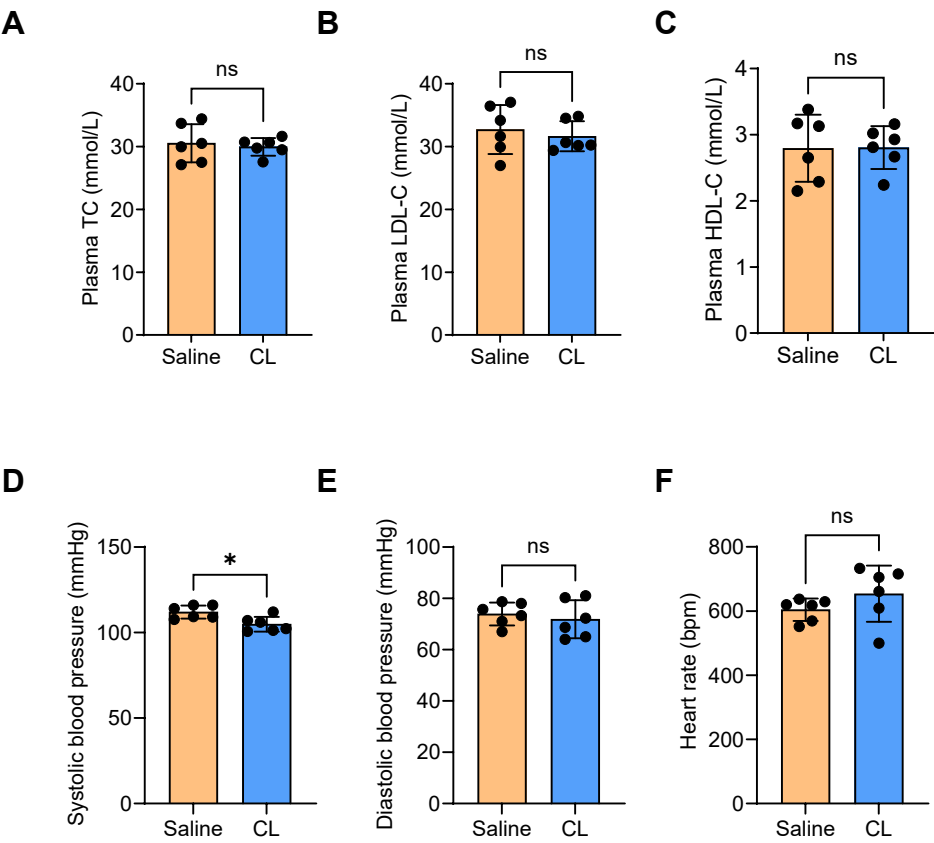

Fig. S7

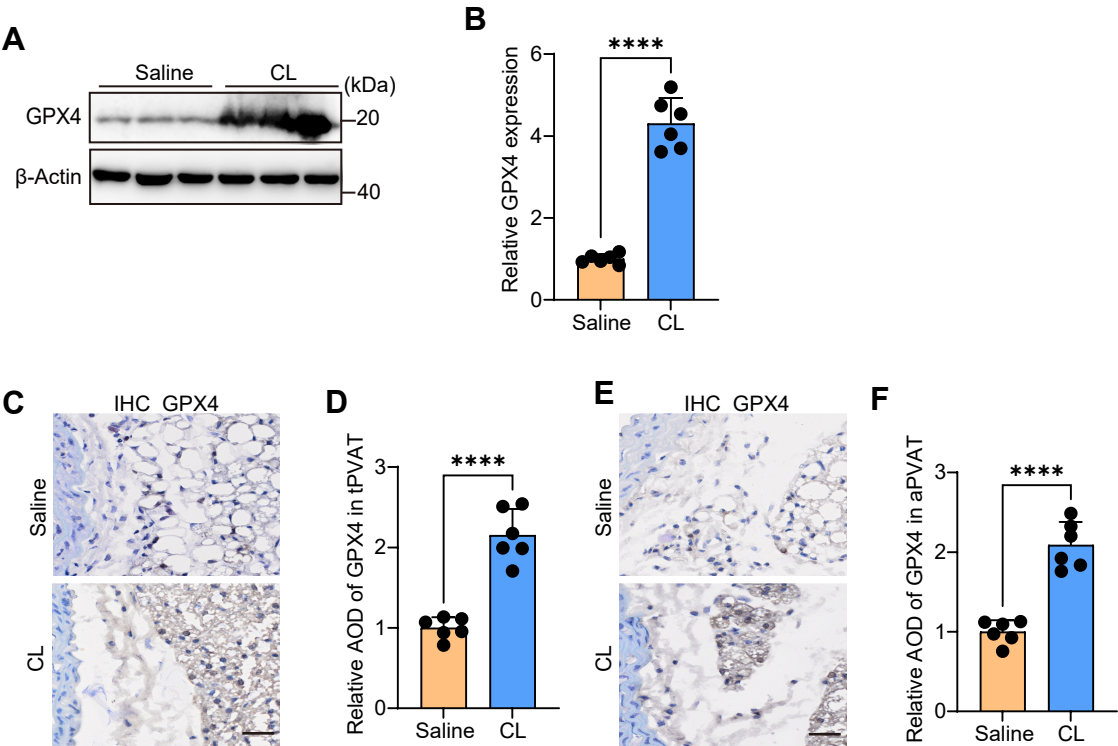

Fig. S8

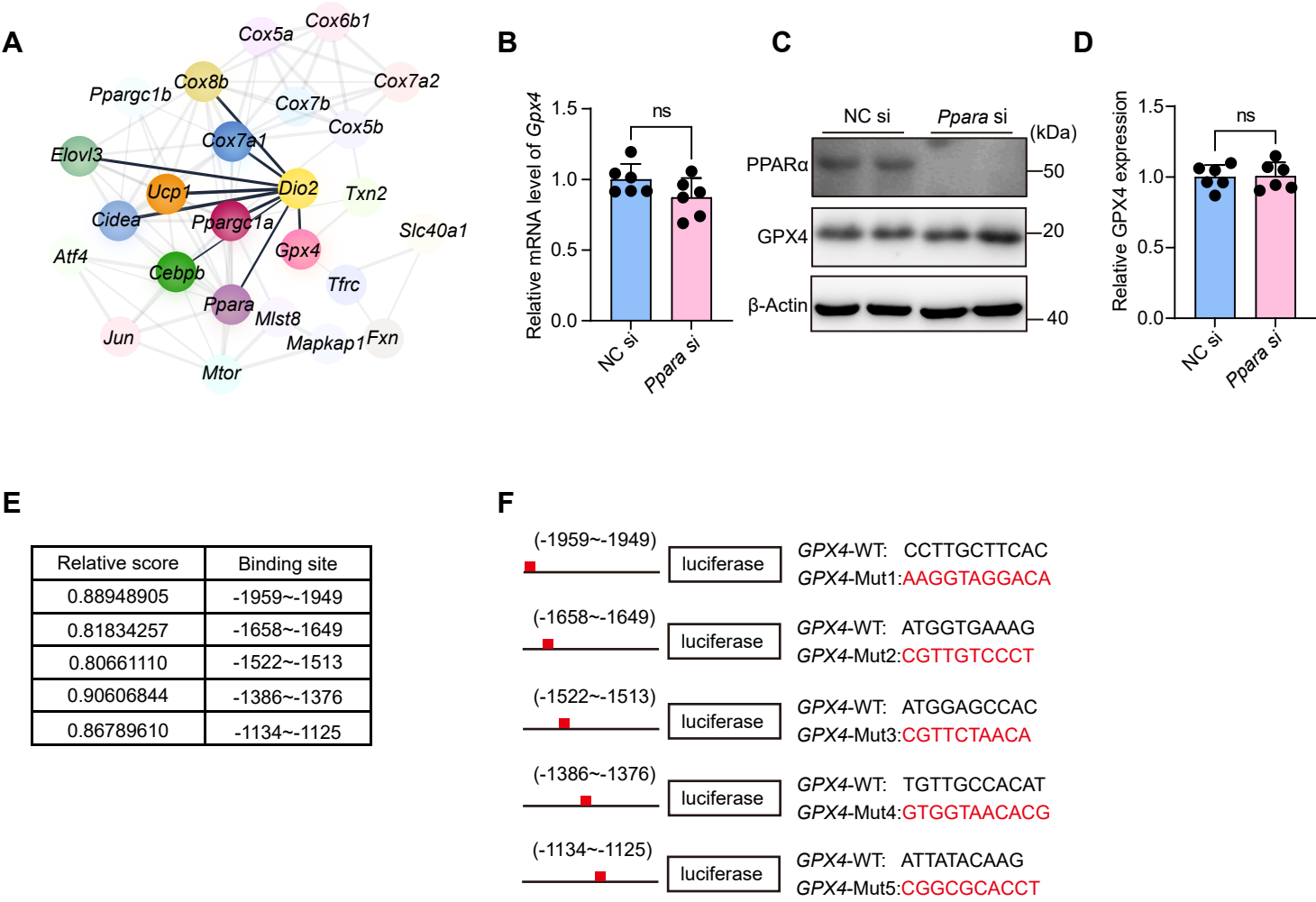

Fig. S9

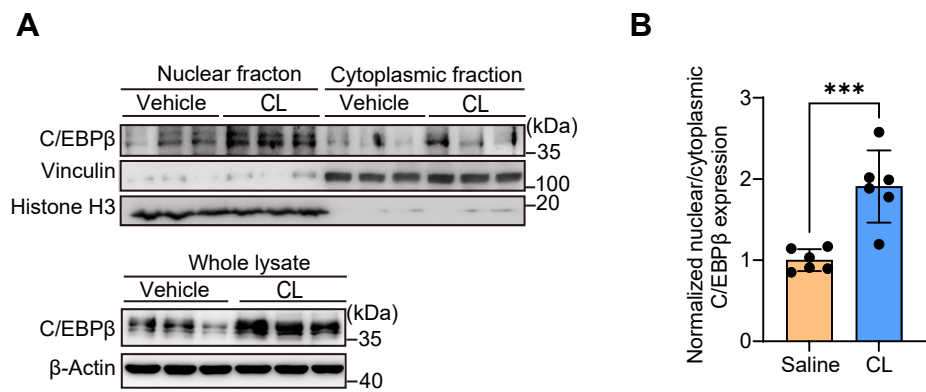

Supplement: Supplementary file 1 — Fig. S1. Gene Set Enrichment Analysis (GSEA) analysis of differentially expressed genes (DEGs) in in PVAT from control mice and atherosclerotic mice. GSEA plot and gene sets for inflammatory response (A), chemokine mediated signaling pathway (B), fatty acid metabolic process (C), and cAMP mediated signaling pathway (D) in PVAT from control mice (Ctrl) and atherosclerotic mice (AS), according to the results of RNA-seq. NES, ES, P-value and FDR value (FDR-corrected P-value) are presented. Fig. S2. GO and KEGG enrichment analysis of differentially expressed genes (DEGs) in high-fat diet (HFD)-treated PVAT and standard fat diet (SFD)-treated PVAT. (A) The GO enrichment bubble plot showing the DEGs of HFD arterial PVAT versus SFD arterial PVAT was determined by GEO dataset analysis (C57BL/6J mice, n = 3 per group). (B) KEGG enrichment bubble plot showing the DEGs of HFD arterial PVAT vs. SFD arterial PVAT was determined by GEO dataset analysis (C57BL/6J mice, n = 3 per group). All raw data were collected from Gene expression omnibus (GEO) database contained SFD-fed or HFD-fed C57BL/6J mice arterial PVAT RNA sequencing samples (GSE28440). Fig. S3. Correlation analyses between ferroptosis-related markers and PVAT dysfunction-related markers in PVAT. (A) Correlation between MDA levels and relative mRNA levels of Il1b. (B) Correlation between MDA levels and relative mRNA levels of Lep. (C) Correlation between GSH/GSSG and relative mRNA levels of Il1b. (D) Correlation between GSH/GSSG and relative mRNA levels of Lep. (E) Correlation between relative mRNA levels of Acsl4 and Il1b. (F) Correlation between relative mRNA levels of Acsl4 and Lep. (G) Correlation between relative mRNA levels of Slc7a11 and Il1b. (H) Correlation between relative mRNA levels of Slc7a11 and Lep. (I) Correlation between relative mRNA levels of Gpx4 and Il1b. (J) Correlation between relative mRNA levels of Gpx4 and Lep. Correlation analyses were conducted using two-tailed Pearson correlation analysis. F [file mmc1.pdf]
