# Supplementary material for: CL-316243 facilitates stable atherosclerotic plaque phenotypes in association with suppression of perivascular adipose tissue ferroptosis via upregulating C/EBPβ
Source: Redox Biol. 2026 Jun 13;95:104259. doi: 10.1016/j.redox.2026.104259 (PMC13279912; doi:10.1016/j.redox.2026.104259)
Supplement: Multimedia component 2 [file mmc2.docx]

**Supplementary Table 1** Primer sequences for quantitative real-time PCR.

| Genes | Forward | Reverse |
| --- | --- | --- |
| *Tnf* | 5'-CAGGCGGTGCCTATGTCTC-3' | 5'-CGATCACCCCGAAGTTCAGTAG-3' |
| *Il1a* | 5'-CGAAGACTACAGTTCTGCCATT-3' | 5'-GACGTTTCAGAGGTTCTCAGAG-3' |
| *Il1b* | 5'-GCAACTGTTCCTGAACTCAACT-3' | 5'-ATCTTTTGGGGTCCGTCAACT-3' |
| *Il6* | 5'-TGGGGCTCTTCAAAAGCTCC-3' | 5'-AGGAACTATCACCGGATCTTCAA-3' |
| *Serpine1* | 5'-TTCAGCCCTTGCTTGCCTC-3' | 5'-ACACTTTTACTCCGAAGTCGGT-3' |
| *Gpx4* | 5'-GCCTGGATAAGTACAGGGGTT-3' | 5'-CATGCAGATCGACTAGCTGAG-3' |
| *Slc27a1* | 5'-GGCACCGTCATCGGATCAG-3' | 5'-CTCCACAGGCAGACCAGAAAA-3' |
| *Nf2* | 5'-GCCATCGCTTCTCGCATGA-3' | 5'-CGCAGTTGAACTCCATCTCGG-3' |
| *Aifm2* | 5'- GCGACCTTCAAGGACAACTTCC-3' | 5'-GCCAGGATAAGATGTGAGAAGGG -3' |
| *Atf4* | 5'-AACCTCATGGGTTCTCCAGCGA-3' | 5'-CTCCAACATCCAATCTGTCCCG-3' |
| *Cebpb* | 5'-GGGCCCTGAGTAATCACTTAAA-3' | 5'-CACTTTAATGCTCGAAACGGAAA-3' |
| *Gapdh* | 5'-AGGTCGGTGTGAACGGATTTG-3' | 5'-TGTAGACCATGTAGTTGAGGTCA-3' |
| *Ccl2* | 5'-TTAAAAACCTGGATCGGAACCAA-3' | 5'-GCATTAGCTTCAGATTTACGGGT-3' |
| *Adgre1* | 5'-TGACTCACCTTGTGGTCCTAA-3' | 5'-CTTCCCAGAATCCAGTCTTTCC-3' |
| *Cd68* | 5'-TGTCTGATCTTGCTAGGACCG-3' | 5'-GAGAGTAACGGCCTTTTTGTGA-3' |
| *Lep* | 5'-TGTGCTGCAGATAGCCAATGA-3' | 5'-TGGAGAAGGCCAGCAGATG-3' |
| *Adipoq* | 5'-AACCCCTGGCAGGAAAGG-3' | 5'-TGAACGCTGAGCGATACACAT-3' |
| *Acsl4* | 5'-  CCTTTGGCTCATGTGCTGGAAC-3' | 5'-GCCATAAGTGTGGGTTTCAGTAC-3' |
| *Slc7a11* | 5'-CTTTGTTGCCCTCTCCTGCTTC-3' | 5'- CAGAGGAGTGTGCTTGTGGACA-3' |
| *Sat1* | 5'- GAGGATGGCTTTGGAGAACACC-3' | 5'- GATACAGCAACTTGCCAATCCATG-3' |
| *Hspb1* | 5'- GCTCACAGTGAAGACCAAGGAAG-3' | 5'- TGAAGCACCGAGAGATGTAGCC-3' |
| *Fth1* | 5'-GCCGAGAAACTGATGAAGCTGC-3' | 5'-GCACACTCCATTGCATTCAGCC-3' |
| *Ucp1* | 5'-GCTTTGCCTCACTCAGGATTGG -3' | 5'-CCAATGAACACTGCCACACCTC -3' |
| *Ppargc1a* | 5'-GAATCAAGCCACTACAGACACCG -3' | 5'-CATCCCTCTTGAGCCTTTCGTG -3' |
